# Derivation and validation of gray-box models to estimate noninvasive in-vivo percentage glycated hemoglobin using digital volume pulse waveform

**DOI:** 10.1038/s41598-021-91527-2

**Published:** 2021-06-09

**Authors:** Shifat Hossain, Shantanu Sen Gupta, Tae-Ho Kwon, Ki-Doo Kim

**Affiliations:** Department of Electronics Engineering, Kookmin University, Seoul, South Korea

**Keywords:** Imaging and sensing, Type 2 diabetes, Physical examination

## Abstract

Glycated hemoglobin and blood oxygenation are the two most important factors for monitoring a patient’s average blood glucose and blood oxygen levels. Digital volume pulse acquisition is a convenient method, even for a person with no previous training or experience, can be utilized to estimate the two abovementioned physiological parameters. The physiological basis assumptions are utilized to develop two-finger models for estimating the percent glycated hemoglobin and blood oxygenation levels. The first model consists of a blood-vessel-only hypothesis, whereas the second model is based on a whole-finger model system. The two gray-box systems were validated on diabetic and nondiabetic patients. The mean absolute errors for the percent glycated hemoglobin (%HbA1c) and percent oxygen saturation (%SpO_2_) were 0.375 and 1.676 for the blood-vessel model and 0.271 and 1.395 for the whole-finger model, respectively. The repeatability analysis indicated that these models resulted in a mean percent coefficient of variation (%CV) of 2.08% and 1.74% for %HbA1c and 0.54% and 0.49% for %SpO_2_ in the respective models. Herein, both models exhibited similar performances (HbA1c estimation Pearson’s R values were 0.92 and 0.96, respectively), despite the model assumptions differing greatly. The bias values in the Bland–Altman analysis for both models were – 0.03 ± 0.458 and – 0.063 ± 0.326 for HbA1c estimation, and 0.178 ± 2.002 and – 0.246 ± 1.69 for SpO2 estimation, respectively. Both models have a very high potential for use in real-world scenarios. The whole-finger model with a lower standard deviation in bias and higher Pearson’s R value performs better in terms of higher precision and accuracy than the blood-vessel model.

## Introduction

Digital volume pulse (DVP) acquisition is an optical method for detecting blood volume variation in tissue. For the detection of blood volume, the tissue is illuminated with light sources of specific wavelengths. The photodetector (PD) and the light sources are placed on the same plane facing the tissue or in two different parallel planes, keeping the tissue sample in between. The photodetector then registers the DVP signal.

DVP signals are generally used to detect time domain properties (e.g., heart rate^1^, respiration rate^2^, etc.) and quantitative parameters (e.g., blood oxygenation^3,4^, hypovolemia and hypervolemia^5^, blood glucose level^6^, etc.) from the human body. Time-domain properties can be estimated with only one wavelength of light, but quantitative properties will require multiple wavelengths of light with some model assumptions. Also, in a previous work, we proposed a new electronic circuit based on an analog filter, that can separate red and green PPG signals, acquire clean PPG signals, and estimate pulse rate (PR) and peripheral capillary oxygen saturation (SpO_2_)^7^.

Diabetes mellitus is a serious metabolic disease that severely affects over 422 million people around the world^8^. Patients with diabetes are very likely to be affected by other serious diseases, such as heart disease, kidney failure, stroke, eye cataracts, and/or sudden mortality. Therefore, diagnosing diabetes is very important in prediabetic stages to prevent the permanent failure of the body sugar control system that results in diabetes. Two methods can be used for diabetes diagnosis: glucose test (random, fasting, or oral) and glycated hemoglobin (HbA1c) test. HbA1c tests perform as well as or better than plasma glucose tests in diabetes diagnosis^9^. Moreover, in an HbA1c test, one can avoid the variability of the plasma glucose in a full day depending on the lifestyle of the examined person.

Many methods are employed to estimate blood glucose and glycated hemoglobin levels. Over the past few decades, many enzymatic and nonenzymatic electrochemical glucose sensors have also been developed^10–15^, but these methods are invasive. In contrast, noninvasive glucose estimation is a comparatively new topic, although some of its implementations using external bodily tissues (skin tissues) and fluids (e.g., saliva and tears) have been reported^16,17^. Implementations of PPG signals for blood glucose level estimation have also been presented^6^.

The four most common methodologies used for HbA1c estimation are immunoassay, ion-exchange high-performance liquid chromatography (HPLC), boronate affinity chromatography, and enzymatic assays^18^. These methodologies require a whole blood sample and are performed by different chemical and/or electrochemical means. However, to date, noninvasive in-vivo research methodologies have not yet been performed to estimate the percent measurement of the %glycated hemoglobin. A noninvasive classification-based solution (classification among diabetic, obese, and normal control groups) has been applied to mice models by measuring hyperglycemia-associated conditions^19^. One study discussed the estimation of in vitro glycated hemoglobin (HbA1c)^20^, but only focused on the PPG sensor design and did not address noninvasive in-vivo estimation methods. Other conference papers, which also focused on the classification of a person’s diabetic status, did not perform estimation of glycated hemoglobin^21,22^. Another paper focused on breath acetone-based HbA1c estimation^23^, but the error rate was very high.

In this study, glycated hemoglobin (HbA1c) is estimated through an optical plethysmographic system. A single white light is transmitted through the fingertip, and the transmitted light waves of different wavelengths are received with three different optical filters on the optical sensor side. This received light wave is called the DVP signal.

The %HbA1c in the blood is estimated along with the %SpO_2_ value using this received DVP signal of multiple wavelengths of light. The DVP signals of three wavelengths are taken to perform this research and estimate the two abovementioned parameters.

Contrary to the related works described above, which mainly focused on categorizing glycemic levels or assessing diabetic status, this study focuses on the percent estimation of in-vivo glycated hemoglobin levels. These percent glycated hemoglobin levels can be used to control the HbA1c levels of normal people, as well as prediabetic and diabetic patients. Furthermore, this study involves DVP signals that are easy to acquire and require low-cost devices. This allows the wearable device to be configured to estimate the glycated hemoglobin levels on-demand or in a continuous manner, noninvasively. Along with all these advantages, the application of this method can be considered a potential low-cost and accurate glycated hemoglobin estimation device.

## Gray-box model

In mathematics and computational models, the gray-box models have a special role. This model can explain how the whole system operates (like a white-box model), and on the other hand, it also corresponds with the practical reference data matched statistically. Therefore, a gray-box model is a combination of theoretical parts, as well as the data-based black-box model. Here, in this study, we develop theoretically based models based on the physiology of blood transportation and glycation of hemoglobin and combined this model with black-box calibration models.

## Finger models and coefficients

Glycated hemoglobin or HbA1c was estimated herein through an optical sensor and transmitter system. Multiple light waves were transmitted through the fingertip, and the transmitted light waves (for a transmissive system) were recorded with an optical sensor. These recorded signals are called the DVP signals.

Using the DVP signal received from multiple light sources, we calculated the percent glycated hemoglobin (%HbA1c) in the blood along with the percent oxygen saturation (%SpO_2_). These two parameters were estimated at the same time; hence, three light sources were required (i.e., 525, 465, and 615 nm denoted by $\lambda_{1}$, $\lambda_{2}$, and $\lambda_{3}$, respectively). According to this physiological basis gray-box model-based approach, any three different wavelengths of light can be chosen. However, these wavelengths were chosen to easily implement these models with a simple color sensor. It is also possible to utilize a mobile camera sensor to record DVP signals.

The location of the DVP signal acquisition (e.g., fingertip, upper and lower wrists, earlobe, etc.) was modeled as a simple mathematical model of only the blood components for the first model and the homogenous mixture of tissues, arterial and venous blood, and water for the second model, which is the whole-finger model. The bones were ignored because we assumed that the bone tissues would not transmit enough light to be detected by the optical sensor. The assumption states the bone as a fixed perfect absorber of light contributing to the DC parts of the signal only. The abovementioned models stated the blood as a homogenous mixture of glycated hemoglobin (HbA1c), oxygenated hemoglobin (HbO), and reduced deoxygenated hemoglobin (HHb).

%HbA1c and %SpO_2_ are described as follows:

1 $$\% HbA1c = \frac{{c_{HbA1c} }}{{c_{HHb} + c_{HbO} + c_{HbA1c} }} \times 100{\text{\% ,}}$$

2 $$\% SpO_{2} = \frac{{c_{HbO} }}{{c_{HHb} + c_{HbO} }} \times 100{\text{\% ,}}$$

where, $c_{HbA1c}$, $c_{HbO}$, and $c_{HHb}$ are the molar concentrations of HbA1c, HbO, and HHb, respectively. The denominator of $\% SpO_{2}$ does not include $c_{HbA1c}$ or any other components because the base for $\% SpO_{2}$ contains only oxygen-bonded hemoglobin cells and hemoglobin cells available for binding with oxygen^24^.

The %Glycated hemoglobin and %Oxygen saturation were estimated using two forms of the finger model. The two models were based on two different hypotheses and described in the following sections.

### Blood-vessel model

**Figure 1:**
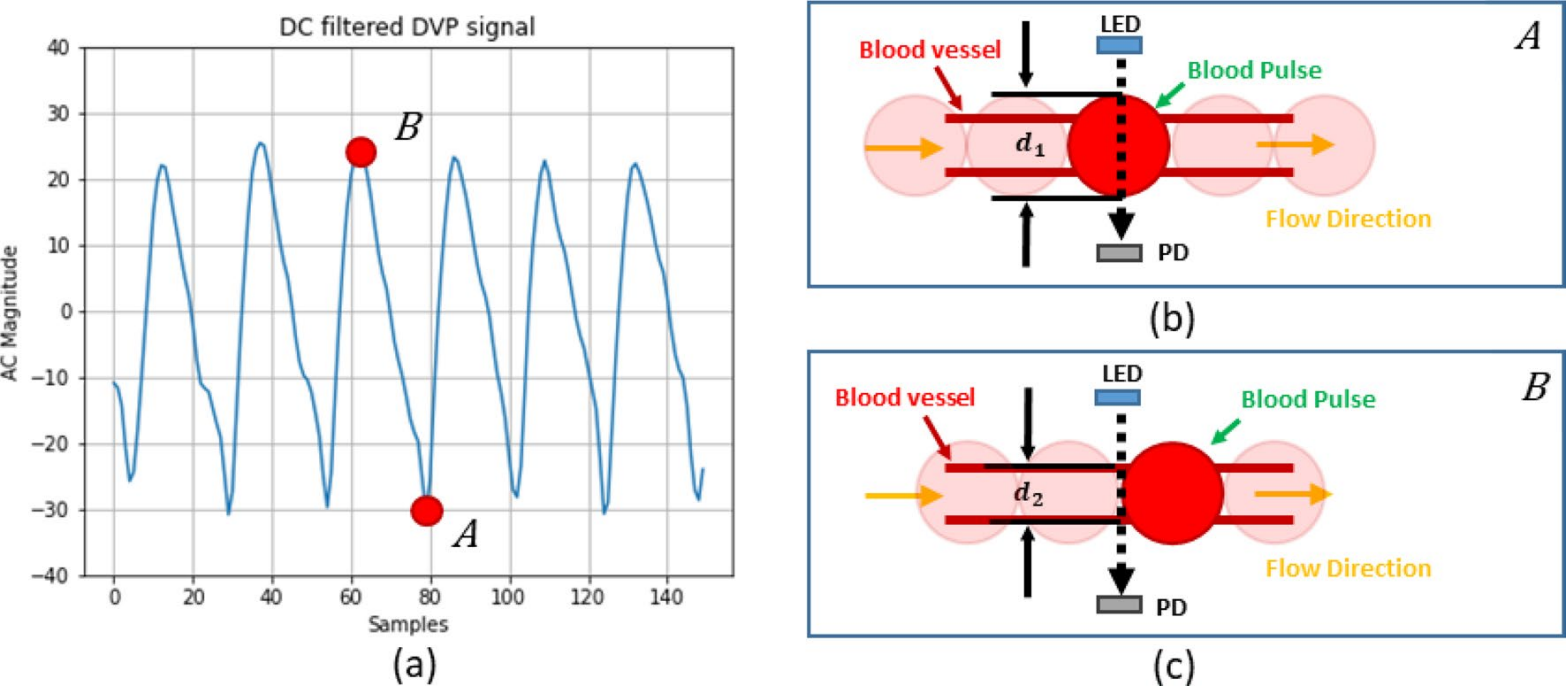
Blood-vessel model illustration with hypothetical blood pulses: (**a**) DVP signal, (**b**) light intensity in the systolic phase, and (**c**) light intensity in the diastolic phase. The variables, $d_{1}$ and $d_{2}$ are the diameter of the blood-vessel when blood pulse enters the blood-vessel and leaves the vessel, respectively. In addition, the photodetector and light emitting diode are denoted as PD and LED, respectively in both (**b,c**).

The first model was built based on the hypothesis that when blood comes into the blood-vessel, the diameter of the vessel slightly expands for the incoming blood volume and reduces the diameter when the blood leaves. Figure 1 depicts the blood-vessel model hypothesis.

The first model only considers blood in the blood vessels; thus, the absorption coefficient of the homogenous mixture of the HbA1c, HbO, and HHb blood components can be calculated as

3 $$C_{a} = \epsilon_{a}^{HbA1c} \left( \lambda \right) \times c_{HbA1c} + \epsilon_{a}^{HbO} \left( \lambda \right) \times c_{HbO} + \epsilon_{a}^{HHb} \left( \lambda \right) \times c_{HHb} ,$$

4 $${\text{Therefore}},\;C_{a} = \mu_{a}^{HbA1c} \left( \lambda \right) + \mu_{a}^{HbO} \left( \lambda \right) + \mu_{a}^{HHb} \left( \lambda \right).$$

In Eq. (3), $C_{a}$ is the total absorption coefficient of the model solution; $\epsilon$ is the molar absorption coefficient $\left[ {{\text{L}}\;{\text{mol}}^{ - 1} \;{\text{cm}}^{ - 1} } \right]$; $c$ is the molar concentration of the attenuator $\left[ {{\text{mol}}\;{\text{L}}^{ - 1} } \right]$. In Eq. (4), $\mu_{a}^{HbA1c}$, $\mu_{a}^{HbO}$, and $\mu_{a}^{HHb}$ are the absorption coefficients, while $\epsilon_{a}^{HbA1c} \left( \lambda \right)$, $\epsilon_{a}^{HbO} \left( \lambda \right)$, and $\epsilon_{a}^{HHb} \left( \lambda \right)$ are the molar absorption coefficients of HbA1c, HbO, and HHb, respectively.

### Whole-finger model

**Figure 2:**
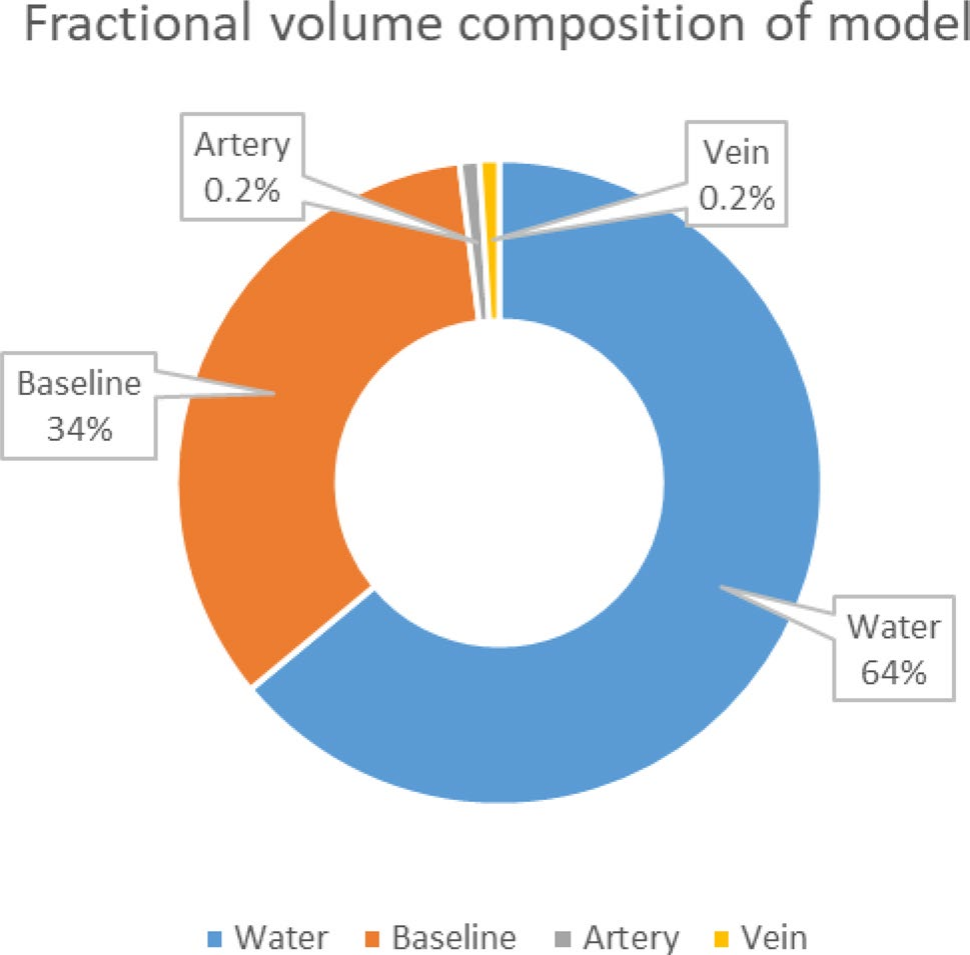
Fractional volume composition of the whole-finger model.

The whole-finger model was constructed based on the homogenous mixture of the lumped finger elements (e.g., dermal tissue, water, and arterial and venous blood). Similar to the previous model, blood is also considered a homogenous mixture of HbA1c, HbO, and HHb hemoglobin cells. Figure 2 illustrates the fractional volume composition of the whole-finger model.

The absorption coefficient of the finger elements can be calculated as

5 $$C_{a} = V_{a} \mu_{a}^{art} \left( \lambda \right) + V_{v} \mu_{a}^{vein} \left( \lambda \right) + V_{w} \mu_{a}^{water} \left( \lambda \right) + \left[ {1 - \left( {V_{a} + V_{v} + V_{w} } \right)} \right]\mu_{a}^{baseline} ,$$

6 $${\text{where}},\;\mu_{a}^{art} = \mu_{a}^{HHb} + P_{HbO}^{art} \left( {\mu_{a}^{HbO} - \mu_{a}^{HHb} } \right) + P_{HbA1c}^{art} \left( {\mu_{a}^{HbA1c} - \mu_{a}^{HHb} } \right),$$

7 $$\mu_{a}^{vein} = \mu_{a}^{HHb} + P_{HbO}^{vein} \left( {\mu_{a}^{HbO} - \mu_{a}^{HHb} } \right) + P_{HbA1c}^{vein} \left( {\mu_{a}^{HbA1c} - \mu_{a}^{HHb} } \right).$$

In Eq. (5), $V_{a}$, $V_{v} ,$ and $V_{w}$ are the partial volume fractions of the artery, vein, and water, respectively; and $\mu_{a}^{art}$, $\mu_{a}^{vein}$, $\mu_{a}^{water}$, and $\mu_{a}^{baseline}$ are the absorption coefficients of the arterial composition, venous composition, water, and lumped dermal skin layer, respectively. In Eqs. (6) and (7), the $\mu_{a}^{HHb}$, $\mu_{a}^{HbO}$, and $\mu_{a}^{HbA1c}$ are not the true absorption coefficients of deoxy-, oxy-, and glycated-hemoglobin. These are the results of the multiplication of the molar absorption coefficient of respective hemoglobin types with whole blood concentration. $P_{HbO}^{art}$, $P_{HbA1c}^{art}$, $P_{HbO}^{vein}$, and $P_{HbA1c}^{vein}$ are the partial molar concentrations of HbO and HbA1c in the artery and vein, respectively. They can be mathematically stated as

8 $$P_{HbO} = \frac{{c_{HbO} }}{{c_{HHb} + c_{HbO} + c_{HbA1c} }},$$

9 $$P_{HbA1c} = \frac{{c_{HbA1c} }}{{c_{HHb} + c_{HbO} + c_{HbA1c} }},$$

10 $$P_{HHb} = 1 - \left( {P_{HbO} + P_{HbA1c} } \right),$$

where $P_{HHb}$ represents the partial molar concentration of HHb(deoxy hemoglobin). Equations (6) and (7) can be easily derived from the following form (refer to Sect. 1 of the Supplementary Document for detailed derivation):

$$\mu_{a} = \epsilon_{a}^{HbA1c} \left( \lambda \right) \times c_{HbA1c} + \epsilon_{a}^{HbO} \left( \lambda \right) \times c_{HbO} + \epsilon_{a}^{HHb} \left( \lambda \right) \times c_{HHb} ,$$

$$\mu_{a} = \left( {c_{Tot} } \right) \times \left( {\epsilon_{a}^{HHb} + P_{HbO} \left( {\epsilon_{a}^{HbO} - \epsilon_{a}^{HHb} } \right) + P_{HbA1c} \left( {\epsilon_{a}^{HbA1c} - \epsilon_{a}^{HHb} } \right)} \right),\;\left[ {{\text{From }}\left( {8} \right), \, \left( {9} \right),{\text{ and }}\left( {{1}0} \right)} \right],$$

where, $$c_{Tot} = c_{HbA1c} + c_{HbO} + c_{HHb} = \frac{150}{{64500}} {\text{mol}}\;{\text{dm}}^{ - 3} .$$

Equations (8) to (10) have the same structure for both artery and vein locations. The molar concentration values in the abovementioned equations were changed according to the location (i.e., artery or vein). The value of the total concentration of blood, $c_{Tot}$ is considered 150/64,500 mol/dm^3^. This value is the typical molar concentration of whole blood. Using the partial molar concentration terminologies (i.e., $P_{HbA1c}$, $P_{HbO} , {\text{and}} P_{HHb}$) as described above, the %HbA1c and %SpO_2_ formulas in Eqs. (1) and (2) can be redefined as follows:

11 $$\% SpO_{2} = \frac{{P_{HbO} }}{{P_{HHb} + P_{HbO} }} \times 100\% ,$$

12 $$\% HbA1c = P_{HbA1c} \times 100\%.$$

## Beer–Lambert law

When blood enters a blood vessel in a certain region, the incident light is absorbed differently compared to the region with no blood because different blood components also absorb light differently. The total absorbance of a homogeneous solution can be mathematically described by the Beer-Lambert Law as follows:

13 $$A = \mathop \sum \limits_{i = 1}^{N} A_{i} = \mathop \sum \limits_{i = 1}^{N} \epsilon_{i} \times c_{i} \times d = - \log \left( {\frac{I}{{I_{0} }}} \right),$$

where *A* is the total absorbance of the solution; $N$ is the number of attenuating species; $\epsilon$ is the molar absorption coefficient $\left[ {{\text{L mol}}^{ - 1} {\text{cm}}^{ - 1} } \right]$; $c$ is the molar concentration of the attenuating species $\left[ {{\text{mol cm}}^{ - 1} } \right];$ and $d$ is the distance traversed by the light beam inside the specimen.

The absorbance of the solution obtained by the Beer-Lambert Law can be directly measured by applying the incident light ($I_{0}$) and measuring the intensity of the light transmitted by the solution ($I$). Therefore, if any homogeneous solution can be represented in the form of (13), it can be solved for an unknown parameter.

**Figure 3:**
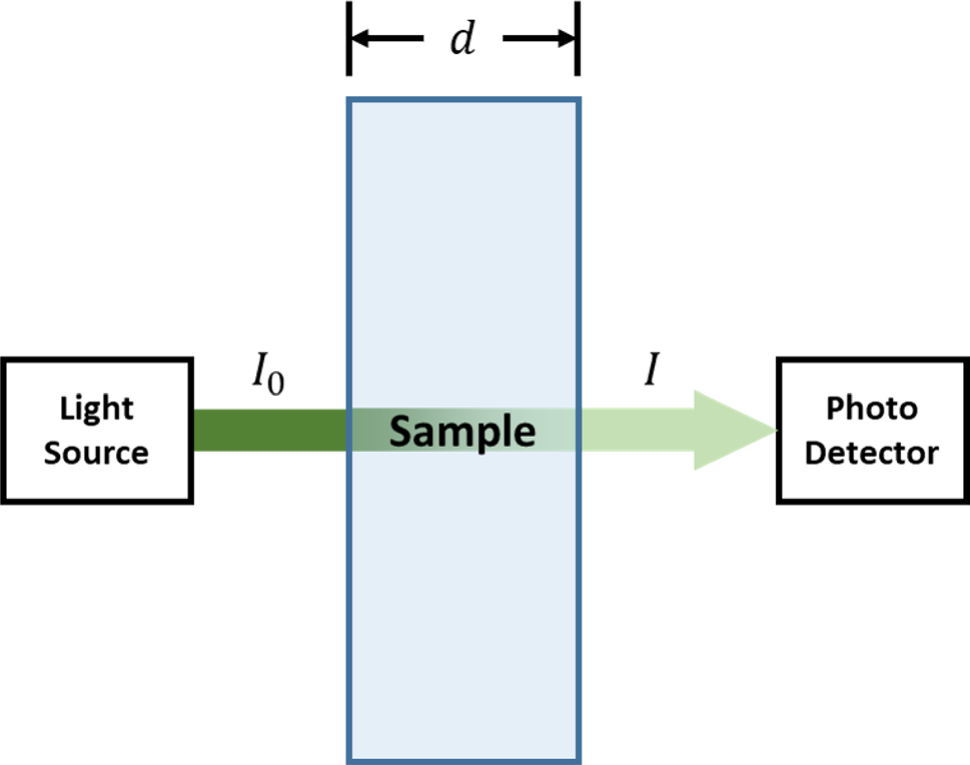
Parameter estimation with the Beer–Lambert law.

The Beer-Lambert Law can be applied to the previously described finger models to obtain the total absorbance of the model solution. The decadic absorption coefficient described in the finger model Eqs. (3) to (5) can be described in terms of absorbance (A) in the following form because the solution is considered homogeneous and will have a uniform absorption along the light traversal path. Figure 3 depicts the parameter estimation utilizing the Beer–Lambert law.

14 $$A = C_{a} d.$$

### Blood-vessel model

The following were obtained when solving the blood-vessel model from Eqs. (3) and (14):

15 $$A = \left( {\epsilon_{a}^{HbA1c} \left( \lambda \right) \times c_{HbA1c} + \epsilon_{a}^{HbO} \left( \lambda \right) \times c_{HbO} + \epsilon_{a}^{HHb} \left( \lambda \right) \times c_{HHb} } \right) \times d.$$

According to this current hypothesis, the molar concentration of the individual components of the model solution will be the same, even when blood comes into the vessels, increasing the volume of the vessel tracts. Therefore, in this assumption, the distance traversed by the light beam inside the finger model will be increased when blood comes in and will be reduced when blood leaves the vessel.

In other words, if absorbance is measured in the two states (i.e., when blood comes in [$A_{1}$] and flows out [$A_{2}$]), the difference between the two states is obtained as

16 $$\delta A = \left( {\epsilon_{a}^{HbA1c} \left( \lambda \right) \times c_{HbA1c} + \epsilon_{a}^{HbO} \left( \lambda \right) \times c_{HbO} + \epsilon_{a}^{HHb} \left( \lambda \right) \times c_{HHb} } \right) \times \delta d,$$

where

$$\delta d = d_{1} - d_{2} , \delta A = A_{1} - A_{2} .$$

For the three light wavelengths (i.e., $\lambda_{1}$, $\lambda_{2}$, and $\lambda_{3}$), Eq. (16) can be written as

17 $$\delta A_{\lambda 1} = \left( {\epsilon_{a}^{HbA1c} \left( {\lambda_{1} } \right) \times c_{HbA1c} + \epsilon_{a}^{HbO} \left( {\lambda_{1} } \right) \times c_{HbO} + \epsilon_{a}^{HHb} \left( {\lambda_{1} } \right) \times c_{HHb} } \right) \times \delta d,$$

18 $$\delta A_{\lambda 2} = \left( {\epsilon_{a}^{HbA1c} \left( {\lambda_{2} } \right) \times c_{HbA1c} + \epsilon_{a}^{HbO} \left( {\lambda_{2} } \right) \times c_{HbO} + \epsilon_{a}^{HHb} \left( {\lambda_{2} } \right) \times c_{HHb} } \right) \times \delta d,$$

19 $$\delta A_{\lambda 3} = \left( {\epsilon_{a}^{HbA1c} \left( {\lambda_{3} } \right) \times c_{HbA1c} + \epsilon_{a}^{HbO} \left( {\lambda_{3} } \right) \times c_{HbO} + \epsilon_{a}^{HHb} \left( {\lambda_{3} } \right) \times c_{HHb} } \right) \times \delta d.$$

From Eqs. (17) to (19), three ratio equations can be obtained, and any two ratio equations can be used to estimate the two unknowns, %HbA1c and %SpO_2_. For convenience, we now define two ratio equations as follows:

20 $$R_{1} = \frac{{\delta A_{\lambda 1} }}{{\delta A_{\lambda 3} }} = \frac{{\epsilon_{a}^{HbA1c} \left( {\lambda_{1} } \right) \times c_{HbA1c} + \epsilon_{a}^{HbO} \left( {\lambda_{1} } \right) \times c_{HbO} + \epsilon_{a}^{HHb} \left( {\lambda_{1} } \right) \times c_{HHb} }}{{\epsilon_{a}^{HbA1c} \left( {\lambda_{3} } \right) \times c_{HbA1c} + \epsilon_{a}^{HbO} \left( {\lambda_{3} } \right) \times c_{HbO} + \epsilon_{a}^{HHb} \left( {\lambda_{3} } \right) \times c_{HHb} }},$$

21 $$R_{2} = \frac{{\delta A_{\lambda 2} }}{{\delta A_{\lambda 3} }} = \frac{{\epsilon_{a}^{HbA1c} \left( {\lambda_{2} } \right) \times c_{HbA1c} + \epsilon_{a}^{HbO} \left( {\lambda_{2} } \right) \times c_{HbO} + \epsilon_{a}^{HHb} \left( {\lambda_{2} } \right) \times c_{HHb} }}{{\epsilon_{a}^{HbA1c} \left( {\lambda_{3} } \right) \times c_{HbA1c} + \epsilon_{a}^{HbO} \left( {\lambda_{3} } \right) \times c_{HbO} + \epsilon_{a}^{HHb} \left( {\lambda_{3} } \right) \times c_{HHb} }}.$$

To represent Eqs. (20) and (21) with the %SpO_2_ and %HbA1c terms, the equations can be simplified with $P_{HbA1c}$, $P_{HbO}$, and $P_{HHb}$ terms from Eqs. (8) to (10). The solved $P_{HbO}$ and $P_{HbA1c}$ terms can then be easily converted to the %SpO_2_ and %HbA1c terms, respectively, using Eqs. (11) and (12).

Thus, applying Eqs. (8) to (10) to Eqs. (20) and (21), we obtain:

22 $$R_{1} = \frac{{P_{HbA1c} \left( {\epsilon^{HbA1c} \left( {\lambda_{1} } \right) - \epsilon^{HHb} \left( {\lambda_{1} } \right)} \right) + P_{HbO} \left( {\epsilon^{HbO} \left( {\lambda_{1} } \right) - \epsilon^{HHb} \left( {\lambda_{1} } \right)} \right) + \epsilon^{HHb} \left( {\lambda_{1} } \right)}}{{P_{HbA1c} \left( {\epsilon^{HbA1c} \left( {\lambda_{3} } \right) - \epsilon^{HHb} \left( {\lambda_{3} } \right)} \right) + P_{HbO} \left( {\epsilon^{HbO} \left( {\lambda_{3} } \right) - \epsilon^{HHb} \left( {\lambda_{3} } \right)} \right) + \epsilon^{HHb} \left( {\lambda_{3} } \right)}},$$

23 $$R_{2} = \frac{{P_{HbA1c} \left( {\epsilon^{HbA1c} \left( {\lambda_{2} } \right) - \epsilon^{HHb} \left( {\lambda_{2} } \right)} \right) + P_{HbO} \left( {\epsilon^{HbO} \left( {\lambda_{2} } \right) - \epsilon^{HHb} \left( {\lambda_{2} } \right)} \right) + \epsilon^{HHb} \left( {\lambda_{2} } \right)}}{{P_{HbA1c} \left( {\epsilon^{HbA1c} \left( {\lambda_{3} } \right) - \epsilon^{HHb} \left( {\lambda_{3} } \right)} \right) + P_{HbO} \left( {\epsilon^{HbO} \left( {\lambda_{3} } \right) - \epsilon^{HHb} \left( {\lambda_{3} } \right)} \right) + \epsilon^{HHb} \left( {\lambda_{3} } \right)}}.$$

The right side of Eq. (13) can be combined with Eqs. (22) and (23) to calculate the ratio equations directly from the received light from the fingertip and obtain

24 $$R_{1} = \frac{{\delta A_{\lambda 1} }}{{\delta A_{\lambda 3} }} = \frac{{\delta \left[ { - \log \frac{I}{{I_{0} }}} \right]_{\lambda 1} }}{{\delta \left[ { - \log \frac{I}{{I_{0} }}} \right]_{\lambda 3} }} = \frac{{\left[ {\log \frac{{I_{0} \left( {d_{1} } \right)}}{{I\left( {d_{1} } \right)}} - \log \frac{{I_{0} \left( {d_{2} } \right)}}{{I\left( {d_{2} } \right)}}} \right]_{\lambda 1} }}{{\left[ {\log \frac{{I_{0} \left( {d_{1} } \right)}}{{I\left( {d_{1} } \right)}} - \log \frac{{I_{0} \left( {d_{2} } \right)}}{{I\left( {d_{2} } \right)}}} \right]_{\lambda 3} }} = \frac{{\left[ {\log \frac{{I\left( {d_{2} } \right)}}{{I\left( {d_{1} } \right)}}} \right]_{\lambda 1} }}{{\left[ {\log \frac{{I\left( {d_{2} } \right)}}{{I\left( {d_{1} } \right)}}} \right]_{\lambda 3} }},$$

25 $${\text{Similarly}},\;R_{2} = \frac{{\delta A_{\lambda 2} }}{{\delta A_{\lambda 3} }} = \frac{{\left[ {\log \frac{{I\left( {d_{2} } \right)}}{{I\left( {d_{1} } \right)}}} \right]_{\lambda 2} }}{{\left[ {\log \frac{{I\left( {d_{2} } \right)}}{{I\left( {d_{1} } \right)}}} \right]_{\lambda 3} }}.$$

Solving Eqs. (22) and (23) for $P_{HbA1c}$ and $P_{HbO}$ yields:

26 $$P_{HbA1c} = \frac{{C_{1} R_{1} + C_{2} R_{2} + C_{3} }}{{C_{4} R_{1} + C_{5} R_{2} + C_{6} }},$$

27 $$P_{HbO} = \frac{{C_{7} R_{1} + C_{8} R_{2} + C_{9} }}{{C_{10} R_{1} + C_{11} R_{2} + C_{12} }}.$$

The coefficients $C_{1}$ to $C_{12}$ are the values obtained after solving Eqs. (22) and (23). The values of these coefficients are given in the “Result and comparison between models” section (“[Sec Sec14]” section) of this manuscript.

### Whole-finger model

As stated earlier, the whole-finger model considers a homogenous mixture of lumped fingertip constitutes. The blood coming inside this model will increase the partial volume fraction of the arterial blood. Simultaneously, the partial volume fractions of the venous blood and water will decrease along with the baseline skin volume fraction. However, note that these transient changes of the venous, water, and skin components were neglected herein for simplicity. The increase in the partial volume fraction of the arterial blood is denoted by ${\Delta }V_{a}$. Therefore, only considering the arterial fraction increment, the absorption coefficient equation becomes

28 $$C_{a} + \Delta C_{a} = \left( {V_{a} + \Delta V_{a} } \right)\mu_{a}^{art} \left( \lambda \right) + V_{v} \mu_{a}^{vein} \left( \lambda \right) + V_{w} \mu_{a}^{water} \left( \lambda \right) + \left[ {1 - \left( {V_{a} + \Delta V_{a} + V_{v} + V_{w} } \right)} \right]\mu_{a}^{baseline} .$$

The change in the absorption coefficient for the change in the arterial blood volume is denoted by ${\Delta }C_{a}$.

Now, subtracting Eq. (5) from Eq. (28), the following is obtained:

29 $$\Delta C_{a} = \Delta V_{a} \left( {\mu_{a}^{art} \left( \lambda \right) - \mu_{a}^{baseline} \left( \lambda \right)} \right).$$

Also, from Eqs. (13) and (14),

30 $$I = I_{0} \times 10^{{ - C_{a} d}} .$$

Equation (30) needs to be differentiated in terms of $C_{a}$ to determine the relation of the physical light intensity with Eq. (29):

31 $$\frac{dI}{{dC_{a} }} = - {\text{ln}}\left( {10} \right)I_{0} d \times 10^{{ - C_{a} d}} .$$

Also,

32 $$\frac{dI}{{dC_{a} }} \approx \frac{{{\Delta }I}}{{{\Delta }C_{a} }}.$$

Equations (31) and (32) yield:

33 $${\Delta }I \approx - {\text{ln}}\left( {10} \right)I_{0} {\Delta }C_{a} d 10^{{ - C_{a} d}} .$$

Now, the AC–DC intensity ratio is generated by the assumption $\frac{{I_{AC} }}{{I_{DC} }} = \frac{{{\Delta }I}}{I}$. The AC part of the signal denotes the pulsatile part of the signal, and vice-versa. Let us then divide Eq. (33) with Eq. (30) and replace ${\Delta }C_{a}$ from Eq. (29):

34 $$\frac{{{\Delta }I}}{I} = - \ln \left( {10} \right)\Delta V_{a} \left( {\mu_{a}^{art} \left( \lambda \right) - \mu_{a}^{baseline} \left( \lambda \right)} \right)d.$$

Similar to the previous model, this equation can be used to make the ratio equations of any two of the three wavelengths. The ratio equations become

35 $$R_{1} = \frac{{\left[ {\frac{{{\Delta }I}}{I}} \right]_{\lambda 1} }}{{\left[ {\frac{{{\Delta }I}}{I}} \right]_{\lambda 3} }} = \frac{{\mu_{a}^{art} \left( {\lambda_{1} } \right) - \mu_{a}^{baseline} \left( {\lambda_{1} } \right)}}{{\mu_{a}^{art} \left( {\lambda_{3} } \right) - \mu_{a}^{baseline} \left( {\lambda_{3} } \right)}},$$

36 $$R_{2} = \frac{{\left[ {\frac{{{\Delta }I}}{I}} \right]_{\lambda 2} }}{{\left[ {\frac{{{\Delta }I}}{I}} \right]_{\lambda 3} }} = \frac{{\mu_{a}^{art} \left( {\lambda_{2} } \right) - \mu_{a}^{baseline} \left( {\lambda_{2} } \right)}}{{\mu_{a}^{art} \left( {\lambda_{3} } \right) - \mu_{a}^{baseline} \left( {\lambda_{3} } \right)}}.$$

Finally, solving Eqs. (35) and (36) gives two equations with the following forms:

37 $$P_{HbA1c}^{art} = \frac{{C_{1} R_{1} + C_{2} R_{2} + C_{3} }}{{C_{4} R_{1} + C_{5} R_{2} + C_{6} }},$$

38 $$P_{HbO}^{art} = \frac{{C_{7} R_{1} + C_{8} R_{2} + C_{9} }}{{C_{10} R_{1} + C_{11} R_{2} + C_{12} }}.$$

The coefficients $C_{1}$ to $C_{12}$ are the values obtained after solving Eqs. (35) and (36). The values of these coefficients are given in the “Result and comparison between models” section (“[Sec Sec14]” section) of this manuscript.

## Data acquisition and processing methodology

A system was designed to acquire the DVP signals from the volunteers and perform experiments on these mathematical models. As the theory for the Beer-Lambert Law states, the nature of the DVP system should be transmissive. Thus, for the fingertip DVP acquisition, the light sources should be on one side of the fingertip, and the sensor should be on the other side. The light rays should pass the fingertip and be received by the sensor. For this reason, a high-intensity light source is required to detect a good-quality signal.

**Figure 4:**
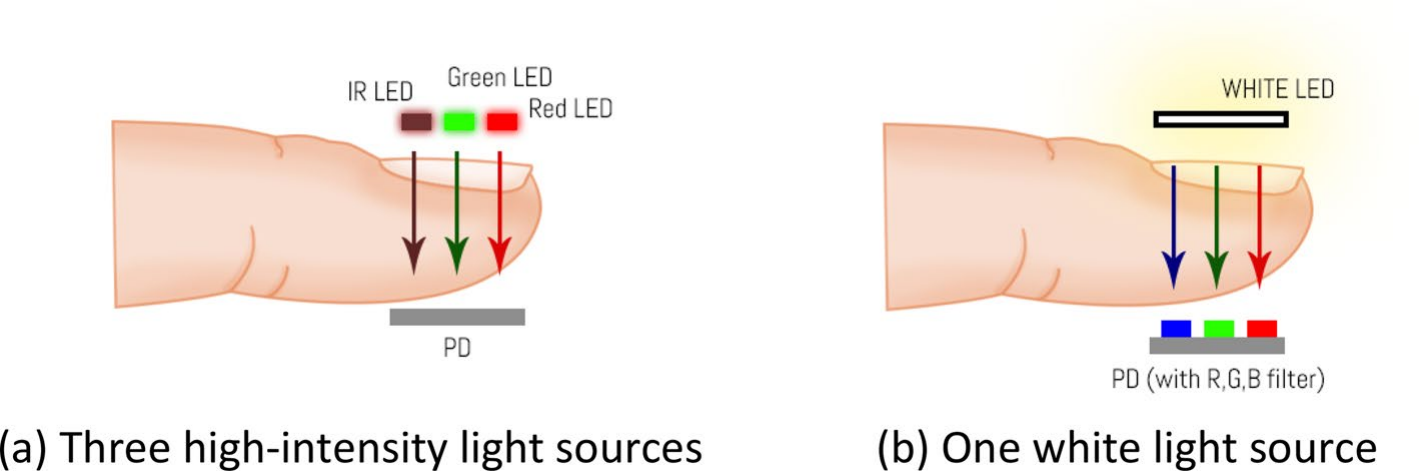
Multiple light sources versus multiple sensor filter systems.

**Figure 5:**
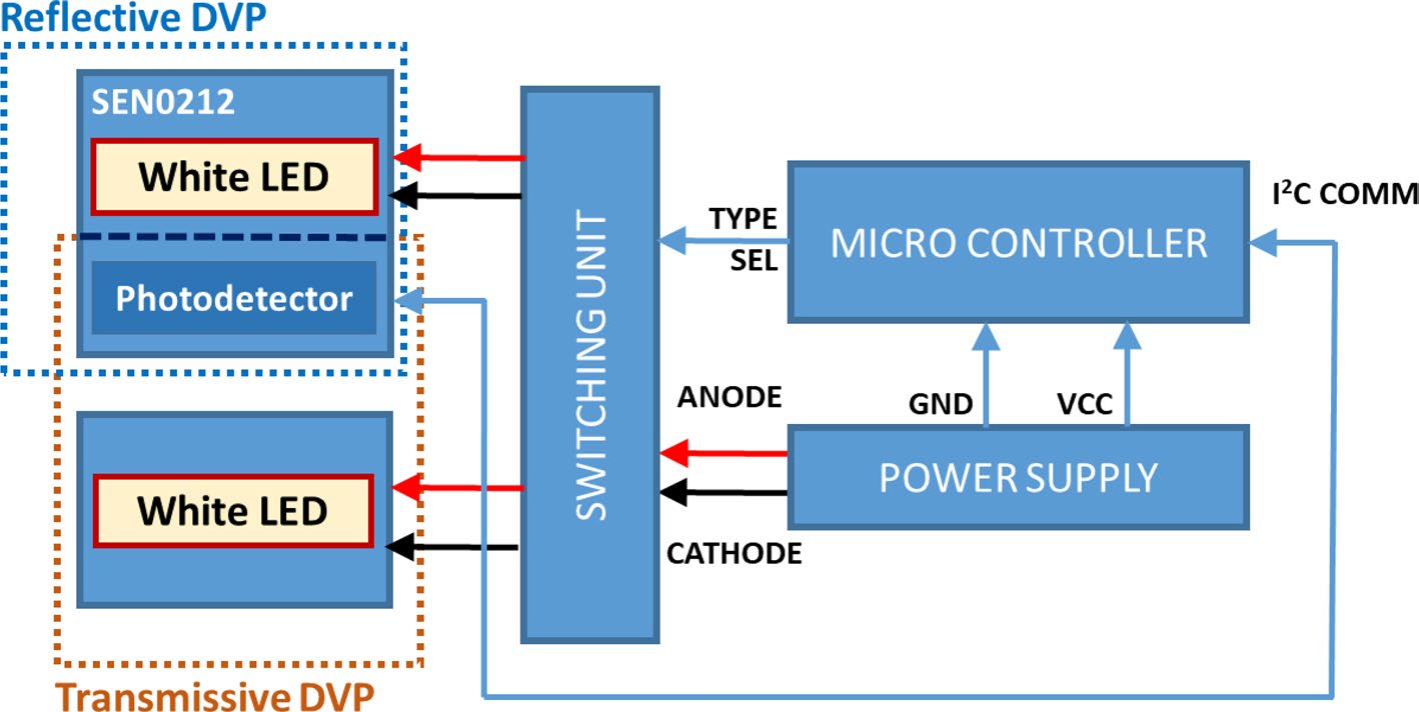
DVP signal acquisition device block diagram.

This model depended on three different wavelengths for the same signal; hence, an RGB color sensor and a white light for the light source were utilized. The color sensor had three different filters on top of the sensor die: blue (465 nm), green (525 nm), and red (615 nm). Clear (i.e., no filter) regions were also present on the sensor. Hence, the space constraint problem in the transmissive DVP system for the light source was solved. Instead of using three high-intensity light sources of different wavelengths, only one white light source and three-wavelength light filters were used on the sensor side (Fig. 4). Figure 5 depicts a basic diagram of the signal acquisition device. In addition to the DVP data, the HbA1c and SpO_2_ reference data were also taken to calibrate and validate these models.

The microcontroller used in this study is Arduino Uno as depicted in Fig. 5. The commercial sensor module DFRobot SEN0212 comprises a color sensor (TCS34725) and a set of four white LEDs. TCS34725 is a highly sensitive sensor with three wavelengths. The wavelengths include 465, 525, and 615 nm. This sensor can run at about 37 Hz sampling rate over the I^2^C protocol.

**Figure 6:**
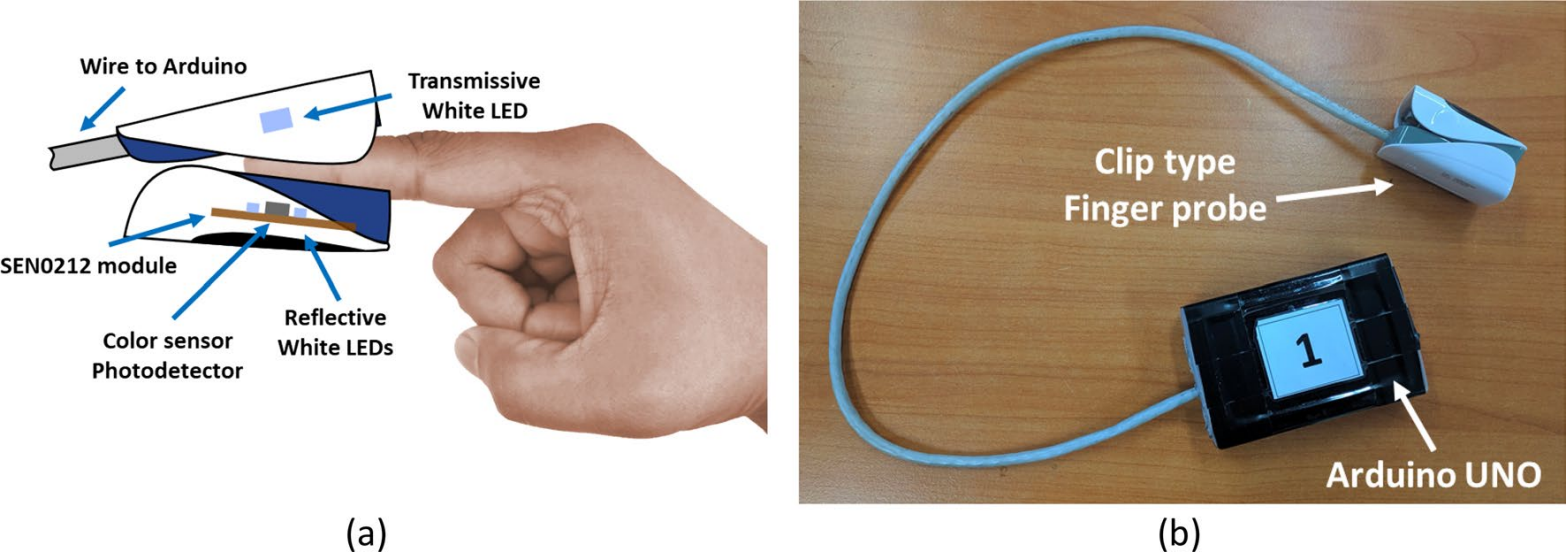
Illustration of (**a**) sensor module-LED arrangement and (**b**) physical image of the device.

The white LEDs with the sensor module are placed around the photodetector. These LEDs are used for recording reflective DVP. For recording transmissive DVP signals, a discrete high-power white LED is attached to the device. The switching unit delivers power to only one of the LEDs (transmissive or reflective) based on the “Type Sel” signal from the microcontroller. The “Type Sel” signal is altered each 1 min to switch the device to change the mode (transmissive or reflective) of the device. For this study, only the transmissive DVP signal was used. The LED and sensor module are attached to a clip-type fingertip device. Figure 6 illustrates the LED-sensor module arrangement in the clip type device and physical device image.

The SEN0212 sensor module (PD and reflective LEDs) is kept in the soft tissue side of the finger, whereas the transmissive white LED is placed over the fingernail as shown in Fig. 6. This arrangement is kept constant for taking data from all the participants.

The Arduino Uno microcontroller is connected to a PC for recording the DVP data. The microcontroller takes the sensor module data and transmits the data via USB serial connection with the PC. This serial data is then saved as comma-separated value (CSV) text files.

These CSV files are then taken into a Python program to preprocess the waveform and calculate the HbA1c and SpO2 values with the equations and calibration procedure described in the subsequent subsections.

The preprocessing of the waveforms includes filtering the waveforms with a second-order Butterworth low-pass filter with a cutoff frequency at 8 Hz. Then, calculate the ratio values with the equations described in the next section. After that, numeric error values and infinite number values are removed from the calculated ratio values. Finally, to remove the effects of noisy signal and miscalculations, data points in the 60% confidence interval (CI) around the mean are taken as the filtered ratio values.

After the preprocessing of the data, the data is fed into the system to evaluate and calibrate the XGBoost models using the leave-one-out-cross-validation (LOOCV) technique. The detailed system description and calibration process are described in the calibration subsection of the following section.

### Human participant ethical compliance

We have compiled ethical regulations for our research methodology from the Institutional Review Board (IRB), Kookmin University, Seoul, Korea. This research was conducted in accordance with the guidelines provided by the IRB, Kookmin University. And we also have obtained informed consent from all the participants for utilizing the data obtained from them, for academic research purposes.

## Result and comparison between models

### Coefficient values on different wavelengths

The data acquisition device used three dominant wavelengths of 465, 525, and 615 nm; thus, the values of the wavelength-dependent parameters for the respective light wavelengths must be evaluated to solve the model equations. These parameters include the molar absorption coefficient of HHb, HbO, and HbA1c given in Table 1 and the absorption coefficient of HHb, HbO, HbA1c, skin baseline, and water given in Table 2. The molar absorption coefficient data of HbA1c were taken from studies by Hossain et al.^25^ and HbO and HHb were taken from Prahl^26^, respectively. The absorption coefficient data of HbA1c, HbO, and HHb were calculated from the molar absorption coefficient multiplied by 150/64,500 mol/L for the whole blood hemoglobin. The absorption coefficient data of the skin baseline and water were taken from studies by Saidi^27^ and Segelstein^28^, respectively.

**Table 1:** Molar absorption coefficient of HbA1c, HbO, and HHb for the respective wavelengths.

Wavelength (nm)	Molar absorption coefficient (${\text{M}}^{ - 1} \;{\text{cm}}^{ - 1}$)		
	HbA1c	HbO	HHb
465	549,024.7353	38,440.2	18,701.6
525	455,139.5677	30,882.8	35,170.8
615	170,555.4218	1166.4	7553.4

**Table 2:** Absorption coefficients of HbA1c, HbO, HHb, skin baseline, and water for the respective light wavelengths.

Wavelength (nm)	Absorption coefficient (cm^−1^)				
	HbA1c	HbO	HHb	Skin baseline	Water
465	1276.8017	89.3958	43.4921	1.6279	0.00020277
525	1058.4641	71.8205	81.7926	1.0966	0.0003927
615	396.6405	2.7126	17.566	0.6552	0.0027167

The absorption coefficient values of HbA1c, HbO, and HHb described in Table 2 are not true absorption coefficients of those parameters. Rather, they are the multiplication of molar absorption coefficients of respective elements with the whole-blood molar concentration.

### Ratio equations with coefficient values

For the blood-vessel model, the following equations were obtained by taking the wavelength ($\lambda$) values as $\lambda_{1} = 525 {\text{nm}}, \lambda_{2} = 465 {\text{nm}},$ and $\lambda_{3} = 615 {\text{nm}}$ and placing the parameter values from Table 1 into Eqs. (22) and (23):

39 $$R_{1} = \frac{{419968.1653 \times P_{HbA1c} - 4288.0 \times P_{HbO} + 35170.8}}{{163002.0218 \times P_{HbA1c} - 6387.0 \times P_{HbO} + 7553.4}},$$

40 $$R_{2} = \frac{{530323.1353 \times P_{HbA1c} + 19738.6 \times P_{HbO} + 18701.6}}{{163002.0218 \times P_{HbA1c} - 6387.0 \times P_{HbO} + 7553.4}}.$$

The following equations were acquired by defining similar wavelength ($\lambda$) values for the whole-finger model and placing the values from Table 2 into Eqs. (35) and (36):

41 $$R_{1} = \frac{{976.6715{ } \times {\text{ P}}_{{{\text{HbA}}1{\text{c}}}} - 9.9721 \times P_{HbO} + 80.696}}{{379.0745{ } \times {\text{ P}}_{{{\text{HbA}}1{\text{c}}}} - 14.8534 \times P_{HbO} + 16.9108}},$$

42 $$R_{2} = \frac{{1233.3096{ } \times {\text{ P}}_{{{\text{HbA}}1{\text{c}}}} + 45.9037 \times P_{HbO} + 41.8642}}{{379.0745{ } \times {\text{ P}}_{{{\text{HbA}}1{\text{c}}}} - 14.8534 \times P_{HbO} + 16.9108}}.$$

### ${{P}}_{{{{HbA}}1{{c}}}}$ and ${{P}}_{{{{HbO}}}}$ equations with coefficient values

At this stage, Eqs. (39) to (42) were solved for $P_{HbA1c}$ and $P_{HbO}$. For the blood-vessel model, Eqs. (39) and (40) were solved, and equations were obtained in the form of Eqs. (26) and (27) with the coefficient values given in Table 3.

**Table 3:** Coefficient values for the $P_{HbA1c}$. and $P_{HbO}$ equations of the blood-vessel model.

*c*_1_	*c*_2_	*c*_3_	*c*_4_	*c*_5_	*c*_6_
13.427	− 9.612	− 38.721	− 330.230	99.169	528.181
*c*_7_	*c*_8_	*c*_9_	*c*_10_	*c*_11_	*c*_12_
− 47.867	− 128.036	539.890	− 330.230	99.169	528.181

Similarly, for the whole-finger model, Eqs. (41) and (42) were solved in the form of Eqs. (37) and (38), with the coefficients given in Table 4.

**Table 4:** Coefficient values for the $P_{HbA1c}$ and $P_{HbO}$ equations of the whole-finger model.

*c*_1_	*c*_2_	*c*_3_	*c*_4_	*c*_5_	*c*_6_
1.398	− 1.030	− 4.122	− 35.720	10.727	57.132
*c*_7_	*c*_8_	*c*_9_	*c*_10_	*c*_11_	*c*_12_
− 4.987	− 14.073	58.636	− 35.720	10.727	57.132

### Clinical dataset information

**Figure 7:**
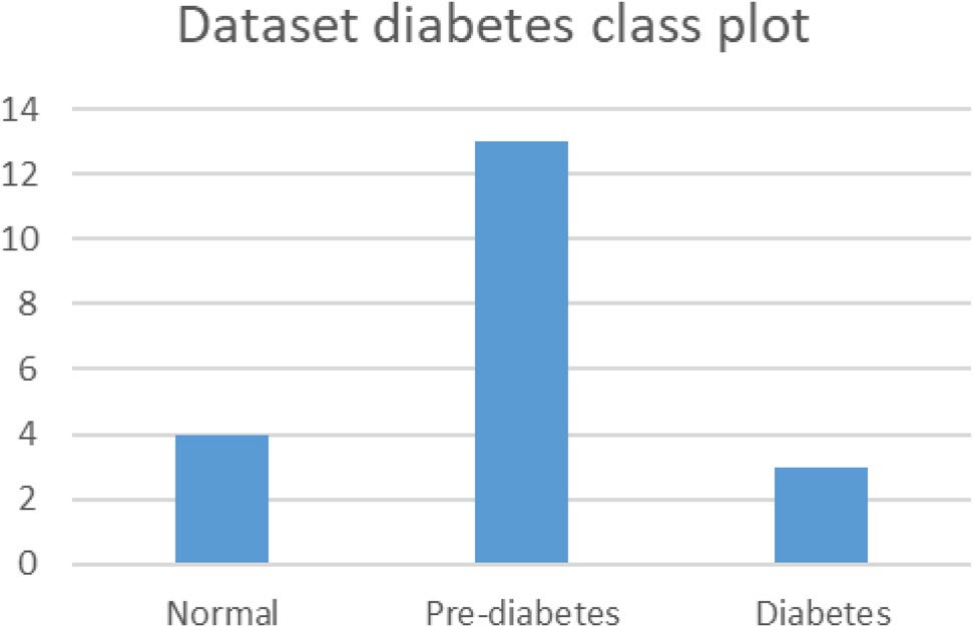
Dataset diabetes class plot.

A small “proof of method” test with 20 participants was conducted to test the hypothesis and model performance. Four volunteers were normal, 13 were in the prediabetic range, and 3 had diabetes (Fig. 7). The age range of the subjects was from 25 to 55 years ($31.6 \pm 10)$. Among the subjects, 5 of them were females and 15 of them were males. The mean and standard deviation (SD) ($Mean \pm SD$) of finger width and BMI of our dataset are $1.30 \pm 0.13$ and $28.86 \pm 3.74$, respectively. Refer to Sect. 8 of the Supplementary Document for complete dataset information.

For each volunteer, 4 min of DVP was recorded, and SpO_2_ data and a National Glycohemoglobin Standardization Program (NGSP) %HbA1c value were measured using an invasive device. The SpO_2_ data were acquired using the Schiller Argus OXM Plus clinical blood oxygenation patient-monitoring device, whereas the invasive %NGSP HbA1c was measured using the BioHermes A1C EZ 2.0 device.

Within the 4 min of recorded DVP signal, 2 min were transmissive DVP signal and the other 2 min were reflective DVP signal. Since the theoretical derivation described above was only on the transmissive DVP signal, the 2 min transmissive DVP signal was used to perform the experiments.

Ethical regulations were compiled for the research methodology from the Institutional Review Board (IRB), Kookmin University, Seoul, Korea. This study was conducted in accordance with the guidelines provided by the IRB, Kookmin University. In addition, prior consent was obtained from all participants in order to utilize the data obtained for academic research purposes.

Any normal and self-reported diabetic volunteers aged 19 to 65 were set to participate in this study. Prospective volunteers were notified to the IRB committee of Kookmin University.

**Figure 8:**
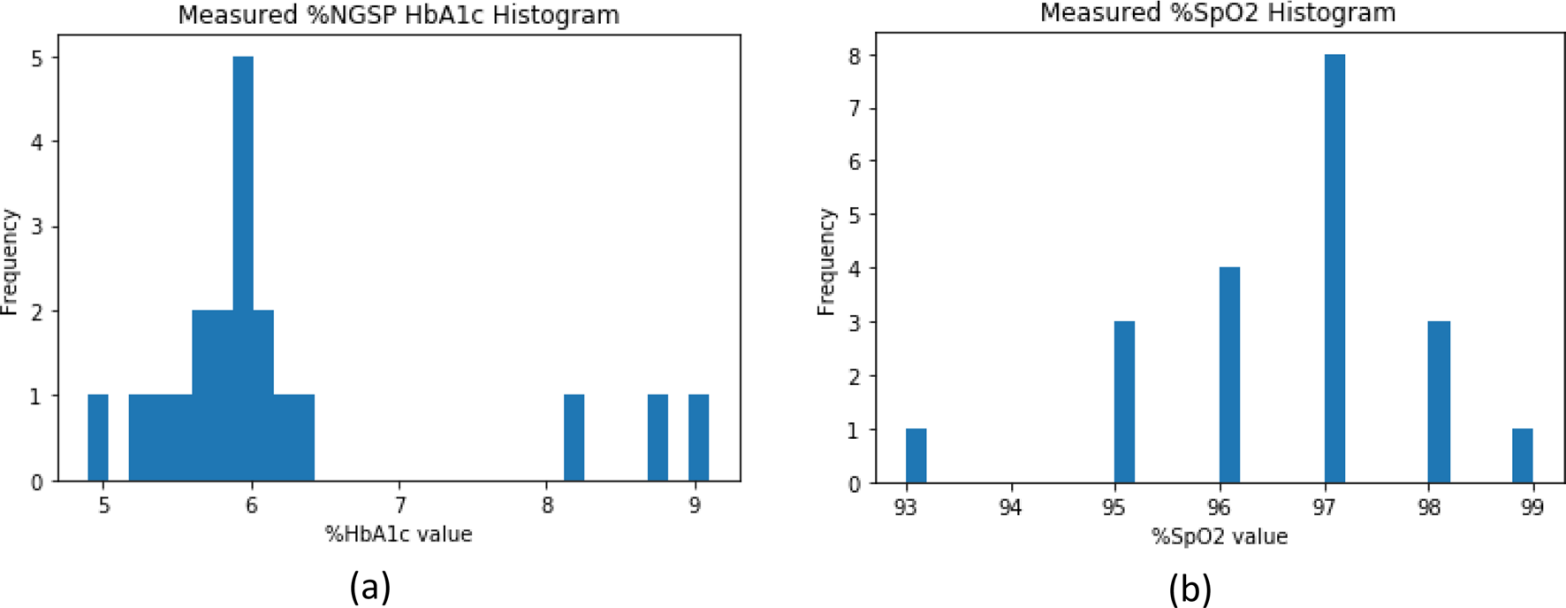
Histogram plot of the measured dataset (**a**) %NGSP HbA1c value and (**b**) %SpO_2_ value.

The volunteers were first checked for any known previous complications that might cause problems either to them or to the experiment. The complications include any record of low blood volume (hypovolemia) and irregular heart rate (tachycardia) within the range of a month. They were then asked to sit idly for approximately 1 to 2 min to stabilize their heart rate. Subsequently, the DVP waveform was recorded from the index finger of the participants with the corresponding devices. The SpO_2_ parameter of a volunteer was recorded in video format from the Argus device display. The volunteers were steady at the time of data acquisition; thus, the variability of blood oxygen saturation was very low. Due to the SpO_2_ invariability, the average of the blood oxygen saturation values was taken for each individual to evaluate the model. Figure 8 depicts the distribution of the %NGSP HbA1c and %SpO_2_ values for the dataset. Table 5 presents the statistics of the %NGSP HbA1c and %SpO_2_ values.

**Table 5:** Statistics of the measured %HbA1c and %SpO_2_ data.

	Min	Max	Mean	Median	SD	Variance	25th Percentile	75th Percentile
%HbA1c	4.9	9.1	6.22	5.9	1.103	1.216	5.7	6.125
%SpO_2_	93	99.0	96.55	97.0	1.322	1.747	96.0	97.0

### Calibration

After dataset creation and data preprocessing, the model was now calibrated with experimental data. These models were based on simple assumptions and processes. Consequently, the models will eventually generate erroneous values without calibration due to model inaccuracy.

The calibration process of this system is performed in two steps. In the first step, the calculated ratio values from the acquired DVP signal are calibrated. In the second step, the calculated HbA1c and SpO_2_ values are calibrated to get more accurate estimations. Each of these calibrations is performed with the XGBoost Regression algorithm. The description of each calibration step is given in the subsequent paragraphs followed by the description of dataset splitting, training–testing, and scoring procedures.

To calibrate each of these models, it was assumed that the measured %NGSP HbA1c and %SPO_2_ values were correct. Based on this assumption, the recorded DVP signal values were first adjusted by calibrating the two ratio values ($R_{1}^{sig}$ and $R_{2}^{sig}$) obtained from the signal amplitudes with the calculated ratio values ($R_{1}^{\prime }$ and $R_{2}^{\prime }$) from reference %HbA1c and %SpO_2_ values. The ratio values from the DVP signal are calculated from light intensity expressions of Eqs. (24) and (25) for blood-vessel model and Eqs. (35) and (36) for the whole-finger model, respectively. The calculated ratio values and deducted from Eqs. (39) and (40) for the blood-vessel model and Eqs. (41) and (42) for the whole-finger model gave the normalized values of the measured reference %HbA1c and %SpO_2_. This step of calibrating the ratio values is crucial because different individuals have different finger widths and different skin and fat layer properties. To reduce the effects of skin, fat layer, and finger width effects on DVP signal amplitudes, this calibration process is applied. In this calibration step, the ratio values from the signal are calibrated to calculated ratio values ($R_{1}^{\prime }$ and $R_{2}^{\prime }$) from Eqs. (39) to (42) with two more features that can compensate for the ratio variability among individuals. The two features are finger width and body mass index (BMI). Therefore, there are four input features, $R_{1}^{sig}$, $R_{2}^{sig}$, finger width, and BMI. The targets are $R_{1}^{\prime }$ and $R_{2}^{\prime }$ for two independent ratio calibrators, respectively. Refer to Sects. 3 and 4 of the Supplementary Document for feature importance metrics for different input features in the ratio calibration step. Furthermore, Sect. 5 of the Supplementary Document contains the analysis of the calibrated ratio values for a different set of input features.

After calibrating the ratio values, the finger model equations were used to estimate the normalized Hba1c and SpO_2_ values. Although these values were close to the reference measurements, these required further calibration to mitigate the model errors (i.e., model inadequacy and propagation errors). This second-level calibration was done on the calculated HbA1c and SpO_2_ values given the reference HbA1c and SpO_2_ as targets, respectively. Refer to Sects. 6 and 7 of the Supplementary Document for a detailed analysis of the impacts of features on the estimation of HbA1c levels. Both calculated HbA1c and SpO_2_ values were provided as the inputs to the calibration model. The reference HbA1c and reference SpO_2_ values were considered as the target values for the respective value calibration models.

The training and testing of these calibration models were performed with leave-one-out-cross-validation (LOOCV) technique. This is a modified K-fold cross-validation technique, in which the number folds are equal to the number of participants in our study. In each fold, the data from one participant is set to test the model, whereas the other participants’ data are provided to train the model. The patient for testing the model is chosen randomly, and each participant’s data are set to be tested exactly once.

**Figure 9:**
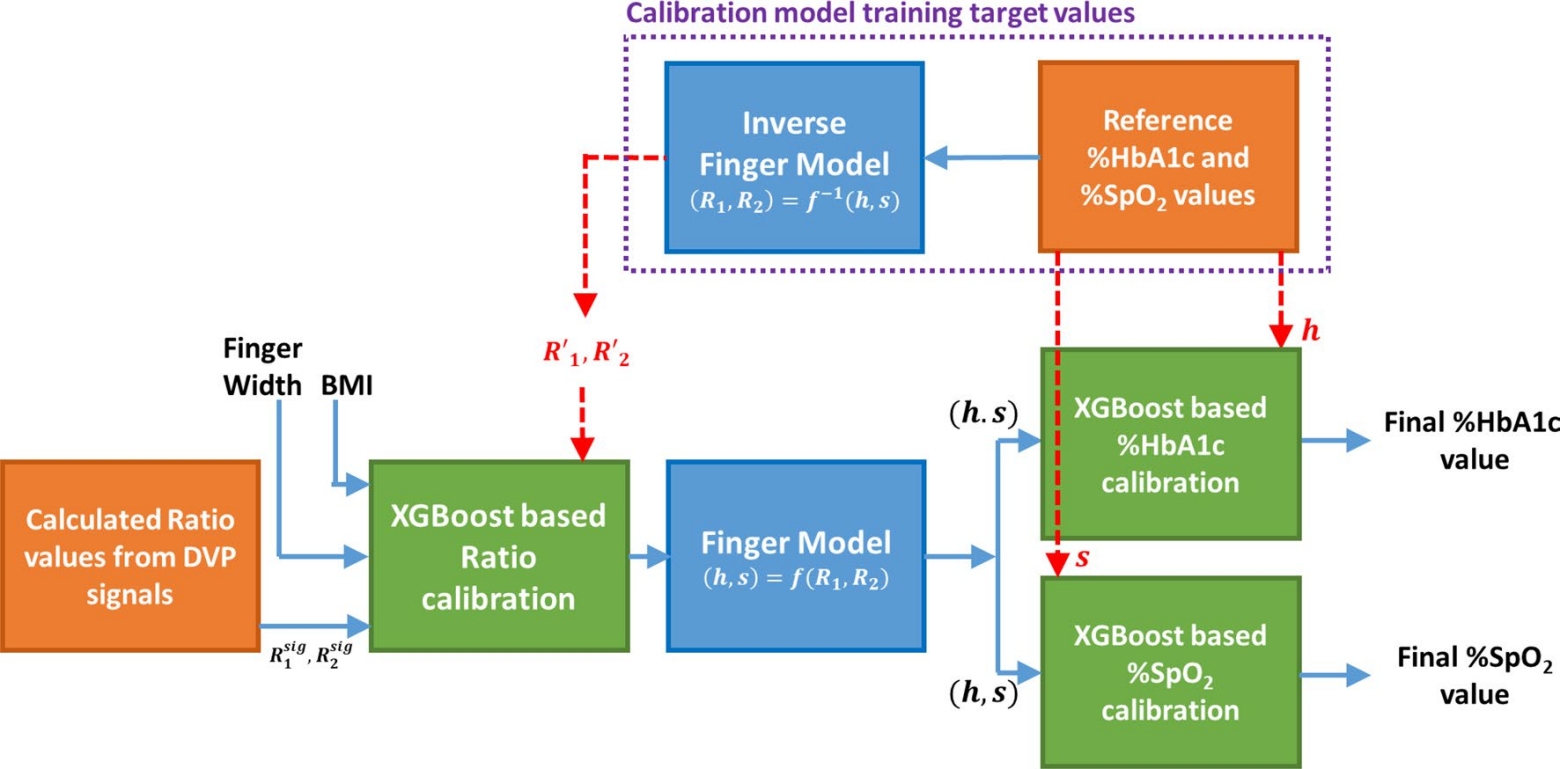
Proposed system overview diagram with calibration blocks. (h = normalized HbA1c, s = normalized SpO_2_). The blue lines indicate the flow of data, and the red dotted lines indicate the target value for training the calibration models. The target values are absent in the testing phase.

To train the XGBoost calibration models in each, the reference %NGSP HbA1c and SpO_2_ values of the training cohort are required (Sect. 2 of the Supplementary Document describes the XGBoost model training parameters). In contrast, the testing of the model is free from reference HbA1c and SpO_2_ values. These test results are used for further processing in the system or given as final estimation results and for scoring the estimated results. The block diagram of the overall system overview with the calibration model blocks is shown in Fig. 9.

In Fig. 9, the orange blocks represent the reference and input data sources, the green blocks represent calibration steps, and the blue blocks represent finger models. The calibrator models’ training target values are drawn with dashed red lines, and the inputs are drawn with solid blue lines. The “Inverse Finger Model” and “Reference %HbA1c and %SpO_2_ values” blocks are only required for the dataset of the training cohort in each fold of the LOOCV. For testing the overall system, the reference blocks are not required. The calibrated ratio values using XGBoost regressor with LOOCV test results are passed to the finger model to estimate the normalized HbA1c and SpO_2_ values. Then these estimated normalized HbA1c and SpO_2_ values are again calibrated and the LOOCV test results are considered as the final estimated %HbA1c and %SpO_2_ values.

### Result deduction

**Figure 10:**
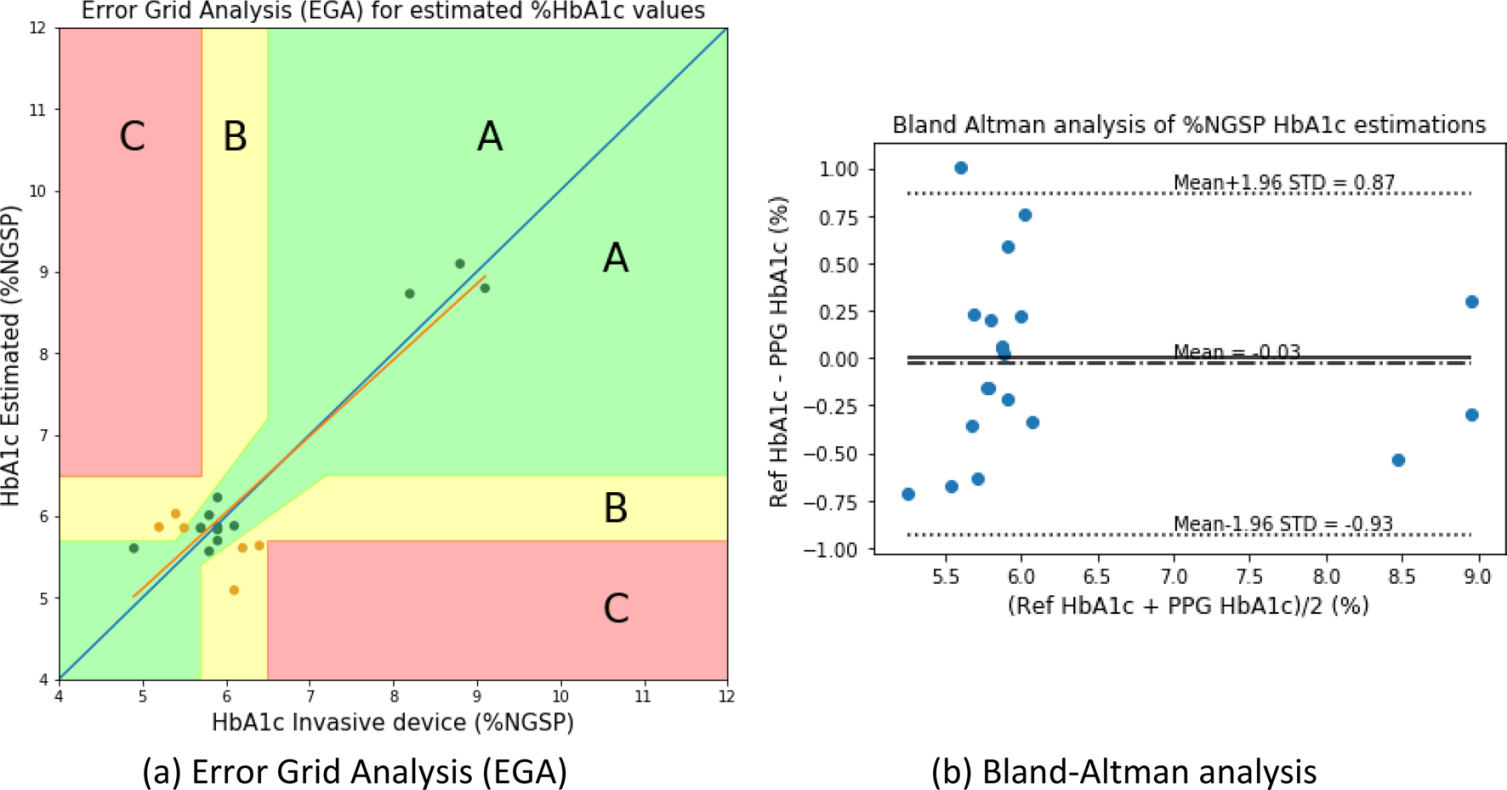
HbA1c Clarke’s error grid analysis (EGA) and Bland–Altman analysis for the blood-vessel model.

**Figure 11:**
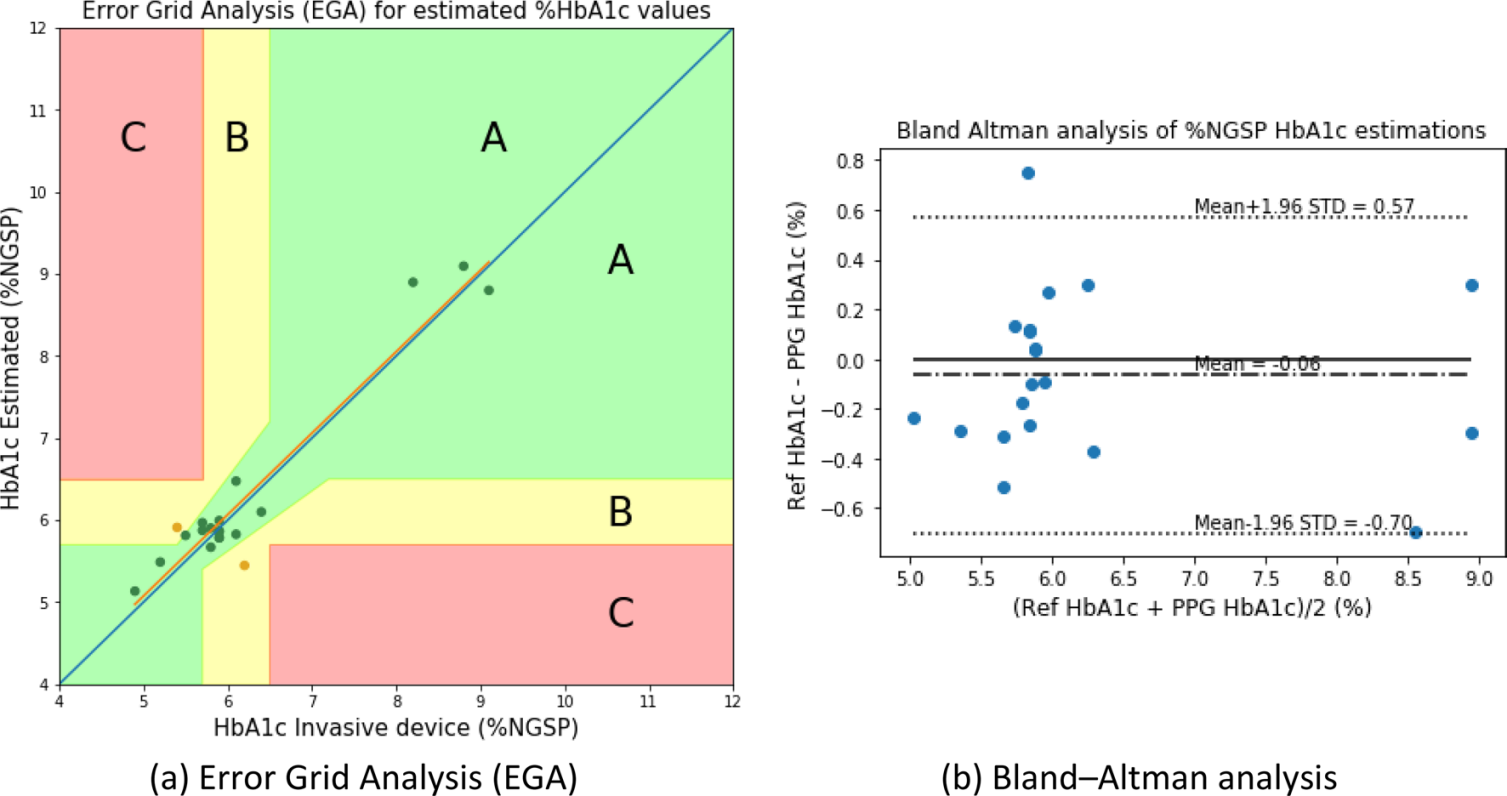
HbA1c Clarke’s error grid analysis (EGA) and Bland–Altman analysis for the whole-finger model.

The following results were obtained with the two models after %HbA1c value calibration: the plot of Clarke’s error grid analysis (EGA)^29,30^ is given with the Bland–Altman analysis in Fig. 10 for the blood-vessel model and Fig. 11 for the whole-finger model.

Figure 10 illustrates that the error grid analysis, with Zone A (clinically accurate data) containing 14 samples (73.68%), Zone B containing 5 samples (26.31%; data outside of 20% of the reference, but would not lead to inappropriate treatment), and Zone C with 0 (0%; data that would lead to uncertain treatment). Figure 11 shows the whole-finger model consisting of 18 (90.0%), 2 (10.0%), and 0 (0%) samples in zones A to C, respectively.

The Bland–Altman analysis indicated that the blood-vessel model provided a bias of – 0.03 ± 0.458, and the limits of agreement (95%; 1.96 SD) ranged from − 0.93 to 0.87. For the whole-finger model, the bias was – 0.06 ± 0.326, and the limits of agreement ranged from − 0.70 to 0.57. The limits of agreement of the whole-finger model were smaller than that of the blood-vessel model.

**Figure 12:**
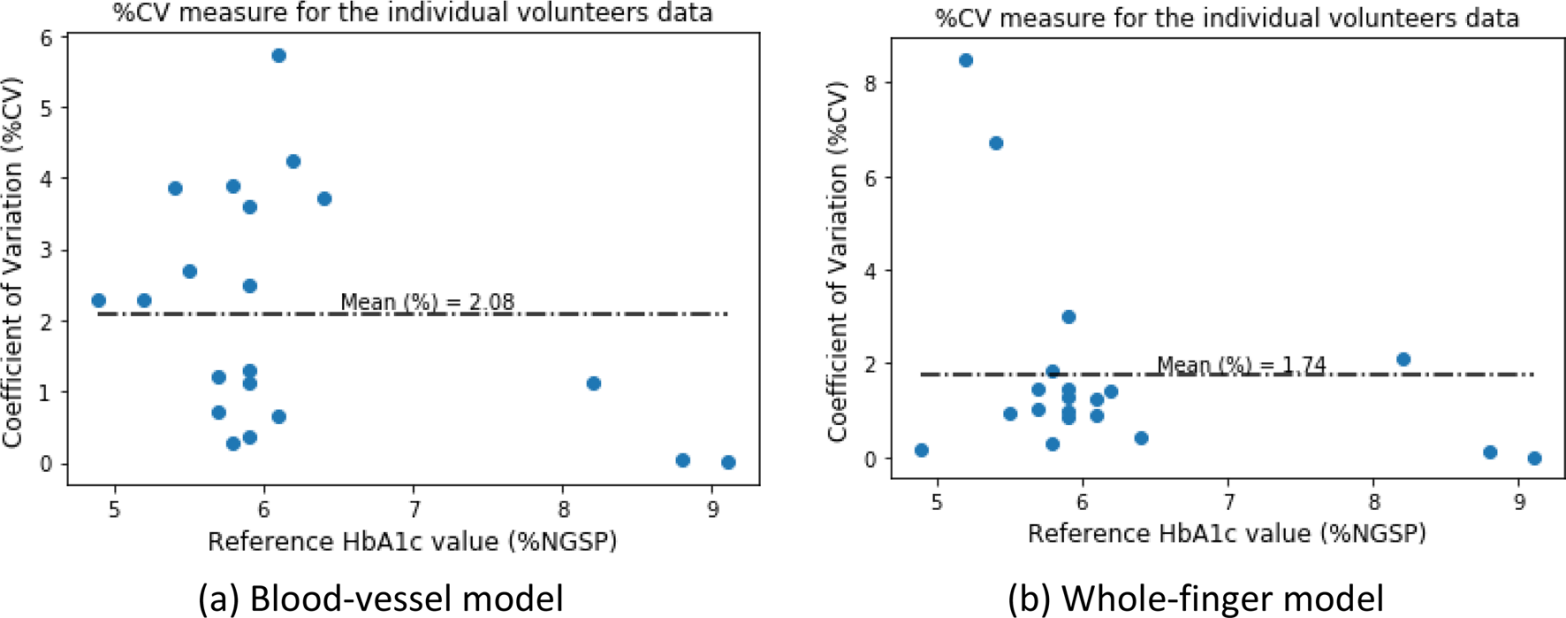
Percent coefficient of variation for the reference and estimated %HbA1c values for both models.

The prediction repeatability was also tested for each patient’s data. Two minutes of the transmissive DVP data were taken; thus, the percent coefficient of variation (%CV) for the predicted %NGSP HbA1c for each data frame (single DVP wave) for each patient is indicated as a measure of the repeatability in the full 2 min of transmissive DVP data. Figure 12 depicts the %CV versus reference %HbA1c data. To estimate the %CV for each participant’s data, all the data frames of a single participant are individually fed into this system to estimate the HbA1c and SpO_2_ values. Then the %CV of the corresponding parameter (i.e., HbA1c or SpO_2_) is calculated with these estimated values.

The %CV plot of all data frames illustrates that the mean %CV was 2.08% for the blood-vessel model and 1.74% for the whole-finger model. These results are very accurate for the repeatability analysis.

The statistical analysis of the estimated and reference %HbA1c data from the blood-vessel model yielded the mean square error (MSE) of 0.211, mean error (ME) of − 0.031, mean absolute deviation (MAD) of 0.375, and root mean square error (RMSE) of 0.459. The Pearson’s R coefficient metric was 0.916.

Similarly, the statistical analysis of the whole-finger model provided 0.110, − 0.065, 0.271, and 0.332 for the MSE, ME, MAD, and RMSE, respectively. The Person’s R coefficient metric was 0.959.

**Figure 13:**
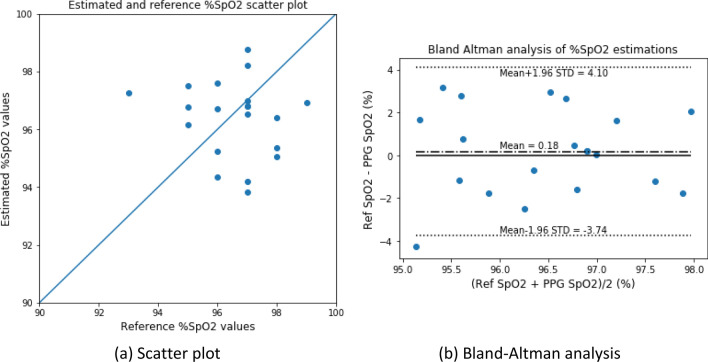
Scatter plot and Bland–Altman analysis of the estimated versus reference (measured) %SpO_2_ values for the blood-vessel model.

**Figure 14:**
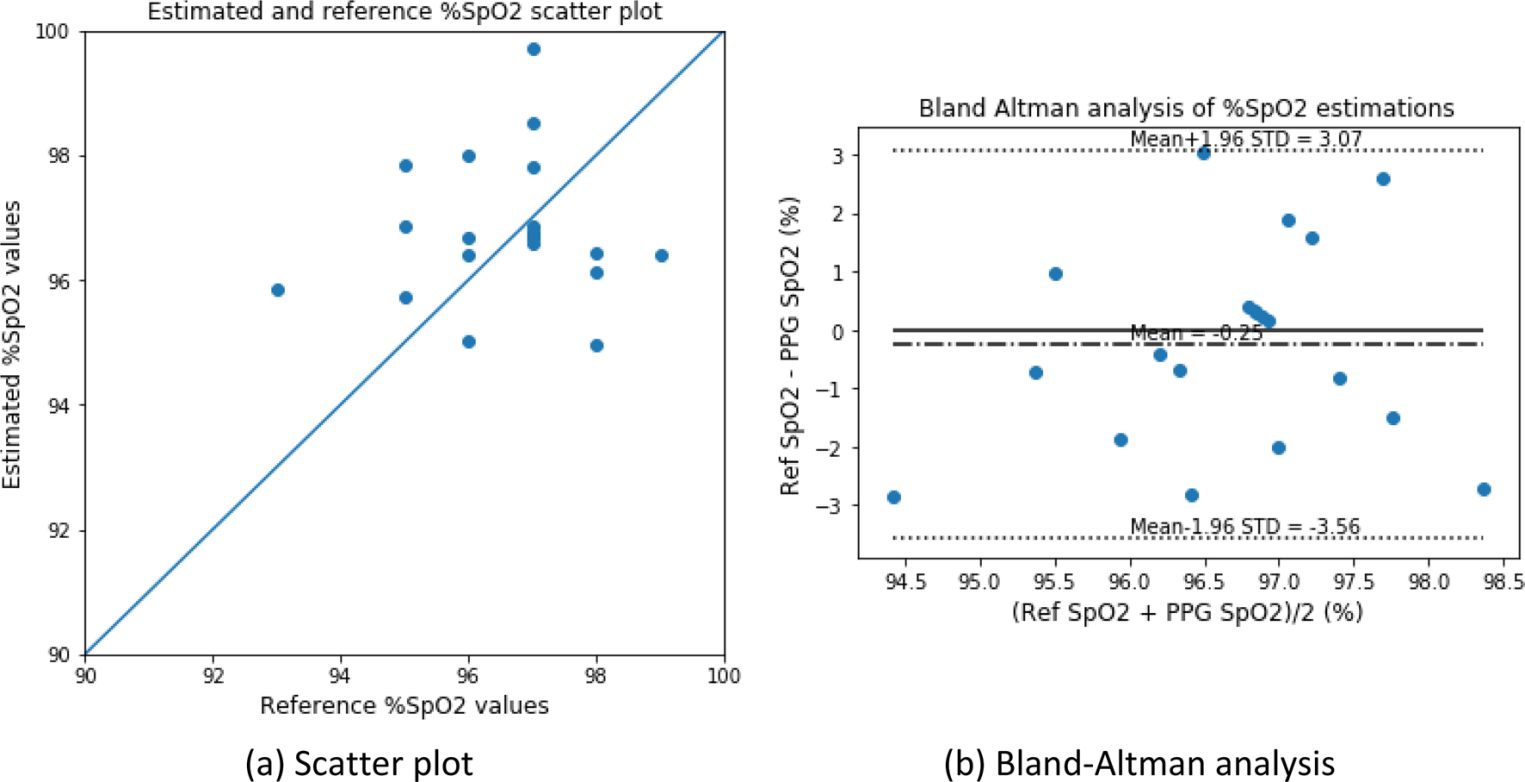
Scatter plot and Bland–Altman analysis of the estimated versus reference (measured) %SpO_2_ values for the whole-finger model.

The estimated %SpO_2_ values were also calibrated and analyzed. Figures 13 and 14 depict the scatter plot and the Bland–Altman analysis of the estimated versus reference %SpO_2_ values for the blood-vessel and whole-finger models, respectively. The Bland–Altman analysis of the %SpO_2_ values showed a bias of $0.178 \pm 2.002$ and – 0.246 ± 1.690 for the blood-vessel and whole-finger models, respectively. The limit of agreement ranged from − 3.74 to 4.10 and − 3.56 to 3.07 for the models, respectively.

**Figure 15:**
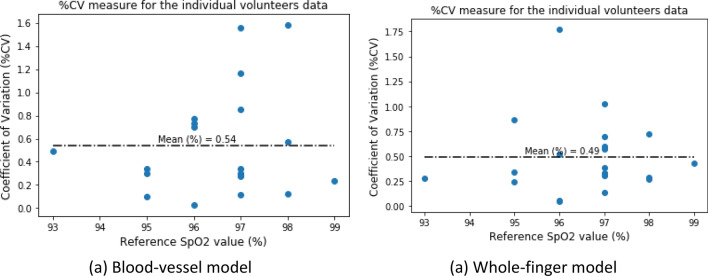
Percent coefficient of variation for the reference and estimated %SpO_2_ values for both models.

For the repeatability analysis of the estimated %SpO_2_ values, the %CV was calculated similarly to the case of %HbA1c values. The maximum %CV was 1.58 and 1.77 for the blood-vessel and whole-finger models, respectively, whereas the mean %CV was 0.54 and 0.49, respectively (Fig. 15).

The statistical analysis with MSE, ME, MAD, and RMSE for the estimated %SpO_2_ values gave 4.038, 0.178, 1.676, and 2.010 for the blood-vessel model and 2.924, − 0.246, 1.395, and 1.710 for the whole-finger model. The reference closeness factor (RCF) was found to be 0.983 and 0.986, respectively, for the two models.

The RCF is a metric to measure the closeness of the reference and estimated values. This metric is calculated with the following equation:

43 $$RCF = \frac{1}{N}\mathop \sum \limits_{i = 1}^{N} \left( {1 - \frac{{\left| {SpO_{2}^{Ref} \left( i \right) - SpO_{2}^{Est} \left( i \right)} \right|}}{100}} \right),$$

where, $N$ is the total number of samples, and $SpO_{2}^{Ref}$ and $SpO_{2}^{Est}$ are the reference and estimated %SpO_2_ levels, respectively.

### Comparison to state-of-the-art methods

Studying the recent studies and state-of-the-art methods regarding noninvasive in-vivo glycated hemoglobin and blood oxygenation estimation, it can be seen that, although there are several studies conducted to estimate blood oxygenation, there are very few studies conducted that classify glycated hemoglobin levels in a noninvasive manner. To the best of the authors’ knowledge, no other research works have been conducted to estimate the percent glycated hemoglobin levels until now. In this section, the two most notable studies on noninvasive HbA1c are compared based on the methodology and advancement.

The most notable study in the field of noninvasive in-vivo classification of glycemic status was conducted by Martín-Mateos et al.^19^. This study was performed on diabetic mouse models to demonstrate the effectiveness of categorizing animals with sustained hyperglycemia under nonglycemic conditions using mm-wave transmission spectroscopy. Although the research illustrated a good approach to categorize glycemic status, it did not estimate the number of glycation products. Similar work on the classification of glycemic states was also conducted by Usman et al.^22^, which utilizes the second derivative of photoplethysmography signals.

Compared to the previous studies, the %HbA1c levels can be accurately estimated by the theoretical derivation of two different models in our study. This HbA1c can be used to control the blood sugar level for prediabetic and diabetic patients, which cannot be performed with the methods mentioned above. Furthermore, the method of the current study makes use of DVP signals, which require low-cost devices, and the signals can be easily acquired. This can enable the construction of wearable devices capable of estimating the percent glycated hemoglobin levels in a continuous manner. Along with all these advantages, the application of this method can be considered as a low-cost instrumentation device for estimating noninvasive glycated hemoglobin having high potential for commercial applications.

In contrast, most commercial pulse oximeters have absolute mean error (or mean absolute deviation) of less than 2% at normal saturation (90–97.5% SpO_2_) and perfusion rate, two-thirds have a standard deviation (SD) of less than 2%, and the other devices have an SD of less than 3%^31^. Another research work, also showed a similar standard deviation of the differences between SaO_2_ and SpO_2_^32^. Most devices had a mean of differences (bias) of up to 2.0%.

Compared to the state-of-the-art blood oxygenation devices, the approach of this study resulted in the SpO_2_ estimation error bias ($Mean \pm SD$) of $0.178 \pm 2.002$ and – 0.246 ± 1.690, for blood-vessel and whole-finger models, respectively. From these metrics, it can be said that the estimation accuracy of SpO_2_ using the system of this study is comparable with the state-of-the-art noninvasive pulse oximeters. The blood-only model provides error metrics similar to the industry-standard oximeters, while the whole-finger model provides better accuracy metrics.

## Discussion

The analysis of the volunteers’ data and results evidently showed comparable performance metrics for both physiological basis gray-box models. The error metrics between the models were similar for the volunteers’ data. The analysis done in these error metrics was based on the mean of a full 2 min of recorded transmissive DVP data. However, looking at the model estimation repeatability, the blood-vessel model has a higher mean %CV than the whole-finger model in both cases of %HbA1c and %SpO_2_ estimations. This can happen due to the model inaccuracy in the blood-vessel model compared to the whole-finger model. The whole-finger model takes into account more parameters of the fingertip, rendering the model more accurate in structure compared to the blood-vessel model. The simpler construction of the blood-vessel model can make it sensitive to the input noise. Therefore, a photosensor with high sensitivity and lower noise margin should be used to utilize the blood-vessel model in practical situations.

It is very important to stress that both models are very simple compared to the physical structure of the fingertip. A physical fingertip differs from person to person in terms of the epidermal, dermal, fat, and muscle layer thickness and volume. The blood volume also differs due to physical effects or abnormalities. These include vasoconstriction, vasodilation, and change in blood pressure and perfusion rate. Also, pressure on the measurement site changes the DVP waveform. These parameters highly affect the calculated ratios because these models cannot consider these uncertainties and might result in high errors. Although this study tried to compensate for the effects of fat tissues, skin types, and finger width of the individuals’ fingers, some unknown parameters can always arise to cause regression errors. However, if these models are calibrated for individuals, they should give a much higher accuracy in the regression analysis as the uncertain parameters are included in the individualistic calibration process.

It is also important to take note of the variance in the measured reference data. The advertised accuracy for the Schiller Argus OXM Plus device (SpO_2_ monitor) was ± 2% for the 70% to 100% range. In contrast, the BioHermes A1C EZ 2.0 device (reference HbA1c device) had an advertised precision of %CV of < 3% in the 4.0 to 6.5%HbA1c range. The device manufacturer, however, did not guarantee the precision above and below the specified range. Our tests showed that the measured %HbA1c value in the range conformed with the advertised precision value, but above 6.5%, the %CV went close to 4.9% (refer to Sect. 9 of Supplementary Document for the test details). These inaccuracies in the reference data led to the error propagation in the model parameters and calibration steps. Taking more patient samples can improve the estimation accuracy greatly for both models.

## Conclusion

In this research, two gray-box models with physiological basis assumptions were deduced to estimate the %HbA1c levels in human blood. The first model only comprised a blood-vessel, whereas the second model considered a full-finger system for absorption effects only. Although these models are simple compared to the realistic fingertip structure, upon validation, this study was able to estimate the %NGSP HbA1c and %SpO_2_ in clinically accurate regions (region A in EGA plots) in most cases, and the estimation was clinically plausible (region B in EGA plots) in the other cases for multiple volunteers’ data. No in-vivo non-invasive studies were previously performed to estimate the percent glycated hemoglobin with digital volume pulse waveform. Therefore, this study is a strong proof of method in this scope.

Some more factors can be considered in future studies. For example, light scattering in biological media, finger structure variability, light sources, and detector properties should be examined to obtain better results. A more controlled calibration can also be performed to reduce the error in the reference data by increasing the data sample size and improving the data purification algorithm.

## Supplementary Information

Supplementary Information.

## Data Availability

The dataset used in this research is available upon a valid request to any of the authors of this research paper.

## Code availability

The codes used in this study are available in Github (https://github.com/ShifatHossain/hba1c_BLM).
