# Supplementary material for: Derivation and validation of gray-box models to estimate noninvasive in-vivo percentage glycated hemoglobin using digital volume pulse waveform
Source: Sci Rep. 2021 Jun 9;11:12169. doi: 10.1038/s41598-021-91527-2 (PMC8190179; doi:10.1038/s41598-021-91527-2)
Supplement: Supplementary file 1 — Supplementary Information. [file 41598_2021_91527_MOESM1_ESM.docx]

**Supplementary Document**

**Derivation and Validation of Gray-Box Models to Estimate Noninvasive *In-vivo* Percentage Glycated Hemoglobin using Digital Volume Pulse Waveform**

Shifat Hossain^1^, Shantanu Sen Gupta^1^, Tae-Ho Kwon^1^, and Ki-Doo Kim^1^*

1. **Explanation of derivation of Eqs. (6) and (7) in the manuscript**

We know –

$\mu_{a}=\epsilon_{a}^{HbA1c}\left( \lambda\right)\times c_{HbA1c}+ \epsilon_{a}^{HbO}\left( \lambda\right)\times c_{HbO}+ \epsilon_{a}^{HHb}\left( \lambda\right)\times c_{HHb}$ (S.1)

Now let,

$c_{Total}=c_{HbA1c}+c_{HbO}+c_{HHb}$ (S.2)

Now from (S.1),

$\mu_{a}=c_{Total}\left( \epsilon_{a}^{HbA1c}\times\frac{c_{HbA1c}}{c_{Total}}+ \epsilon_{a}^{HbO}\times\frac{c_{HbO}}{c_{Total}}+ \epsilon_{a}^{HHb}\times\frac{c_{HHb}}{c_{Total}} \right)$ (S.3)

From (8), (9), and (10) in the manuscript,

$\mu_{a}=c_{Total}\left( \epsilon_{a}^{HbA1c}\times P_{HbA1c}+ \epsilon_{a}^{HbO}\times P_{HbO}+ \epsilon_{a}^{HHb}\times P_{HHb} \right)$ (S.4)

$\mu_{a}=c_{Total}\left( \epsilon_{a}^{HbA1c}\times P_{HbA1c}+ \epsilon_{a}^{HbO}\times P_{HbO}+ \epsilon_{a}^{HHb}\times\left( 1-P_{HbO}-P_{HbA1c} \right) \right)$ (S.5)

$\mu_{a}=c_{Total}\left( P_{HbA1c}\left( \epsilon_{a}^{HbA1c}-\epsilon_{a}^{HHb} \right)+ P_{HbO}\left( \epsilon_{a}^{HbO}-\epsilon_{a}^{HHb} \right)+ \epsilon_{a}^{HHb} \right)$ (S.6)

Now let,

$\mu_{a}^{HbA1c}= c_{Total}\epsilon_{a}^{HbA1c}$ (S.7)

$\mu_{a}^{HbO}= c_{Total}\epsilon_{a}^{HbO}$ (S.8)

$\mu_{a}^{HHb}= c_{Total}\epsilon_{a}^{HHb}$ (S.9)

So, applying (S.7), (S.8), and (S.9) in (S.6),

$\mu_{a}=P_{HbA1c}\left( \mu_{a}^{HbA1c}-\mu_{a}^{HHb} \right)+ P_{HbO}\left( \mu_{a}^{HbO}-\mu_{a}^{HHb} \right)+ \mu_{a}^{HHb}$ (S.10)

This equation (S.10) is given in Eqs. (6) and (7) in the manuscript for artery and vein.

1. **XGBoost Regressor parameters in ratio and HbA1c-SpO_2_ value calibration steps**

For the XGBoost regression model, the default parameters were used. More optimum calibration results can be possible if the hyperparameters are tuned with cross-validation techniques after the current research status (proof of method). The values of the most common model parameters are provided below.

1. **Learning rate:** 0.3
2. **Minimum split loss (gamma):** 0
3. **Maximum depth:** 6
4. **Number of estimators:** 100
5. **Objective function:** Squared Error (reg:suqarederror)
6. **Ratio calibration feature importance (weight)**

**Weight feature importance** is the number of times a feature is used to split the data across all trees. Independent models were trained to estimate R1 and R2 values for both blood-vessel and whole finger models.

**All features (R1, R2, finger width, BMI):**

| **Model** | **Feature** | | | |
| --- | --- | --- | --- | --- |
|  | **R1** | **R2** | **Finger Width** | **BMI** |
| **Blood-vessel R1** | 0.53 | 0.25 | 0.08 | 0.13 |
| **Blood-vessel R2** | 0.51 | 0.23 | 0.11 | 0.16 |
| **Whole-finger R1** | 0.51 | 0.26 | 0.09 | 0.13 |
| **Whole-finger R2** | 0.50 | 0.23 | 0.11 | 0.16 |

**Three features (R1, R2, and finger width):**

| **Model** | **Feature** | | | |
| --- | --- | --- | --- | --- |
|  | **R1** | **R2** | **Finger Width** | **BMI** |
| **Blood-vessel R1** | 0.54 | 0.37 | 0.09 |  |
| **Blood-vessel R2** | 0.55 | 0.37 | 0.08 |  |
| **Whole-finger R1** | 0.53 | 0.38 | 0.08 |  |
| **Whole-finger R2** | 0.54 | 0.38 | 0.08 |  |

**Three features (R1, R2, and BMI):**

| **Model** | **Feature** | | | |
| --- | --- | --- | --- | --- |
|  | **R1** | **R2** | **Finger Width** | **BMI** |
| **Blood-vessel R1** | 0.53 | 0.25 |  | 0.22 |
| **Blood-vessel R2** | 0.52 | 0.23 |  | 0.25 |
| **Whole-finger R1** | 0.51 | 0.27 |  | 0.22 |
| **Whole-finger R2** | 0.51 | 0.22 |  | 0.27 |

**Two features (R1 and R2):**

| **Model** | **Feature** | | | |
| --- | --- | --- | --- | --- |
|  | **R1** | **R2** | **Finger Width** | **BMI** |
| **Blood-vessel R1** | 0.60 | 0.40 |  |  |
| **Blood-vessel R2** | 0.61 | 0.39 |  |  |
| **Whole-finger R1** | 0.59 | 0.41 |  |  |
| **Whole-finger R2** | 0.58 | 0.42 |  |  |

1. **Ratio calibration feature importance (gain)**

**Gain feature importance** is the average gain across all splits, in which the feature is used. ‘Gain’ implies the relative contribution of the corresponding feature to the model calculated by taking each feature’s contribution for each tree in the model.

**All features (R1, R2, finger width, BMI):**

| **Model** | **Feature** | | | |
| --- | --- | --- | --- | --- |
|  | **R1** | **R2** | **Finger Width** | **BMI** |
| **Blood-vessel R1** | 0.02 | 2.57e-5 | 0.94 | 0.13 |
| **Blood-vessel R2** | 0.03 | 2.08e-5 | 0.92 | 0.05 |
| **Whole-finger R1** | 0.03 | 5.17e-5 | 0.94 | 0.03 |
| **Whole-finger R2** | 0.04 | 4.32e-5 | 0.92 | 0.04 |

**Three features (R1, R2, and finger width):**

| **Model** | **Feature** | | | |
| --- | --- | --- | --- | --- |
|  | **R1** | **R2** | **Finger Width** | **BMI** |
| **Blood-vessel R1** | 0.028 | 0.004 | 0.967 |  |
| **Blood-vessel R2** | 0.027 | 0.005 | 0.968 |  |
| **Whole-finger R1** | 0.027 | 0.004 | 0.969 |  |
| **Whole-finger R2** | 0.027 | 0.004 | 0.969 |  |

**Three features (R1, R2, and BMI):**

| **Model** | **Feature** | | | |
| --- | --- | --- | --- | --- |
|  | **R1** | **R2** | **Finger Width** | **BMI** |
| **Blood-vessel R1** | 0.16 | 8.26e-5 |  | 0.84 |
| **Blood-vessel R2** | 0.18 | 3.10e-5 |  | 0.82 |
| **Whole-finger R1** | 0.17 | 1.28e-4 |  | 0.83 |
| **Whole-finger R2** | 0.20 | 4.76e-5 |  | 0.80 |

**Two features (R1 and R2):**

| **Model** | **Feature** | | | |
| --- | --- | --- | --- | --- |
|  | **R1** | **R2** | **Finger Width** | **BMI** |
| **Blood-vessel R1** | 0.73 | 0.27 |  |  |
| **Blood-vessel R2** | 0.73 | 0.27 |  |  |
| **Whole-finger R1** | 0.72 | 0.28 |  |  |
| **Whole-finger R2** | 0.72 | 0.28 |  |  |

1. **R1-R2 plots for different calibrations**

**Inverse finger model R1-R2 plot for the blood-vessel model from reference HbA1c and SpO_2_ values:**

This plot is generated from Eqs. (39) and (40),

$R_{1} = \frac{419968.1653 \times P_{HbA1c} - 4288.0 \times P_{HbO} + 35170.8}{163002.0218 \times P_{HbA1c} - 6387.0 \times P_{HbO} + 7553.4}$ (39)

$R_{2} = \frac{530323.1353 \times P_{HbA1c} + 19738.6 \times P_{HbO} + 18701.6}{163002.0218 \times P_{HbA1c} - 6387.0 \times P_{HbO} + 7553.4}$ (40)


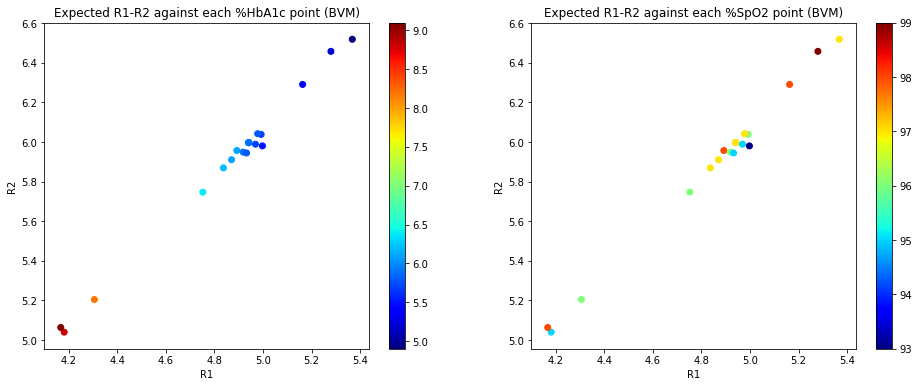


Figure 1: R1-R2 plot for reference %HbA1c and %SpO_2_ values in the blood-vessel model.

**Inverse finger model R1-R2 plot for whole-finger model from reference HbA1c and SpO_2_ values:**

This plot is generated from Eqs. (41) and (42),

$R_{1}=\frac{976.6715 \times P_{HbA1c} - 9.9721 \times P_{HbO} + 80.696}{379.0745 \times P_{HbA1c} - 14.8534 \times P_{HbO} + 16.9108}$ (41)

$R_{2}=\frac{1233.3096 \times P_{HbA1c} + 45.9037 \times P_{HbO} + 41.8642}{379.0745 \times P_{HbA1c} - 14.8534 \times P_{HbO} + 16.9108}$ (42)


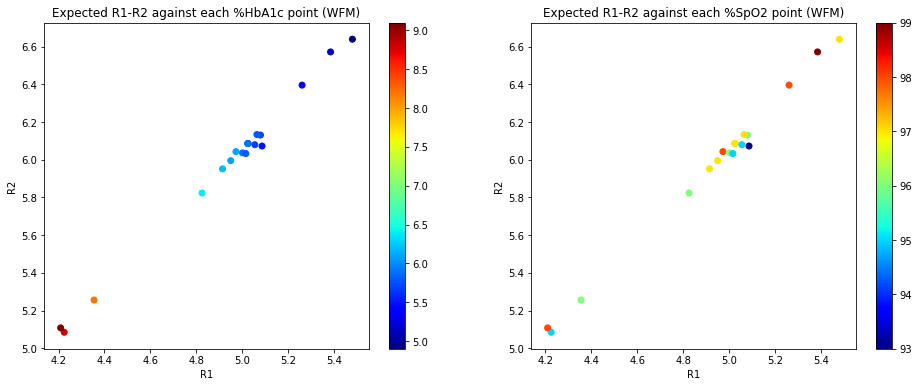


Figure 2: R1-R2 plot for reference %HbA1c and %SpO_2_ values in whole-finger model.

From Fig. 1 and 2, it can be seen that the R1-R2 values for both models are almost similar .

Then we train the calibration models with the R1-R2 values calculated from the DVP signals as input and the reverse calculated R1-R2 from the reference values as the target.

Figure 3 depicts the calculated R1-R2 points for all the volunteers’ DVP data. And Fig. 4 illustrates the average of R1-R2 values per person.


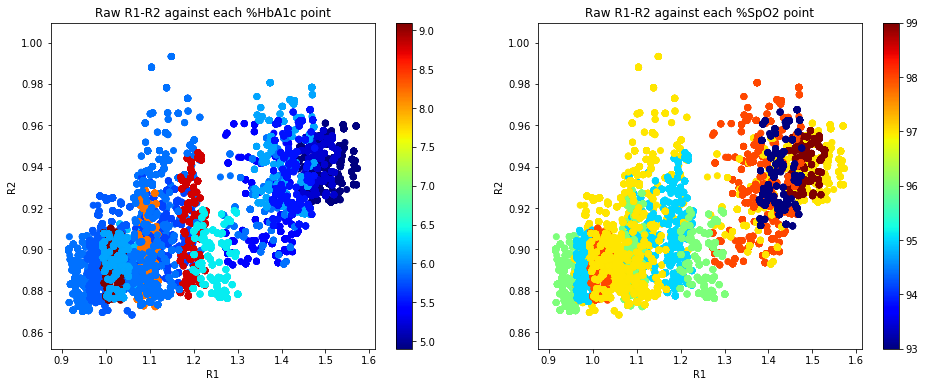


Figure 3: Calculated R1-R2 values for all volunteers’ DVP data.


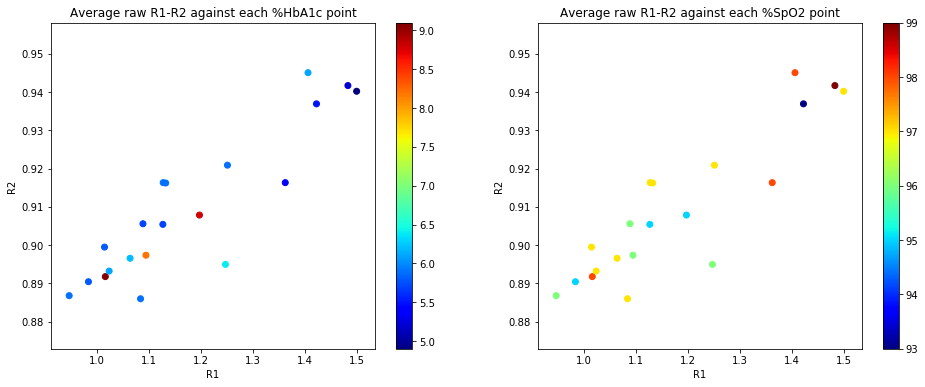


Figure 4: Calculated average R1-R2 values for each volunteers’ DVP data.

Now for calibrating these calculated R1-R2 values, four input features were selected – R1, R2, Finger width, and BMI. The calibrated R1-R2 values are given below as Figs. 5-8 for different feature input combinations in the blood vessel model.


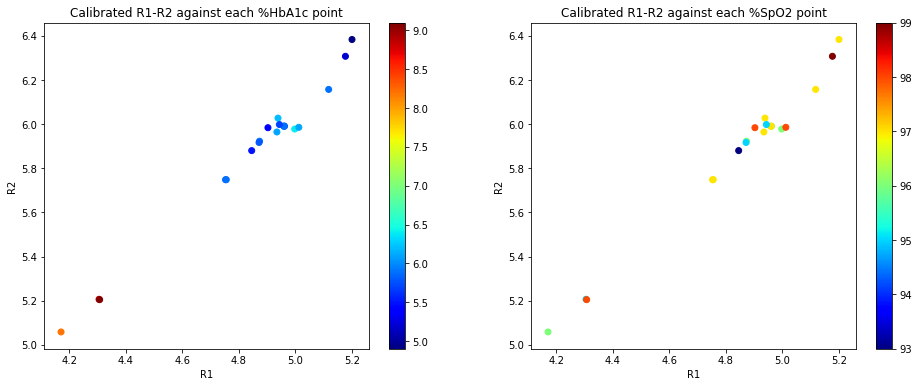


Figure 5: Calibrated R1-R2 for blood vessel model having calculated R1-R2, finger width, and BMI as input features.


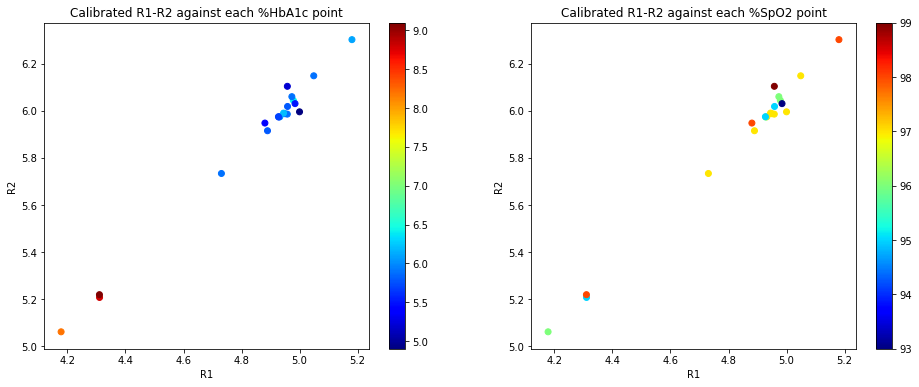


Figure 6: Calibrated R1-R2 for blood vessel model having calculated R1-R2 and finger width as input features.


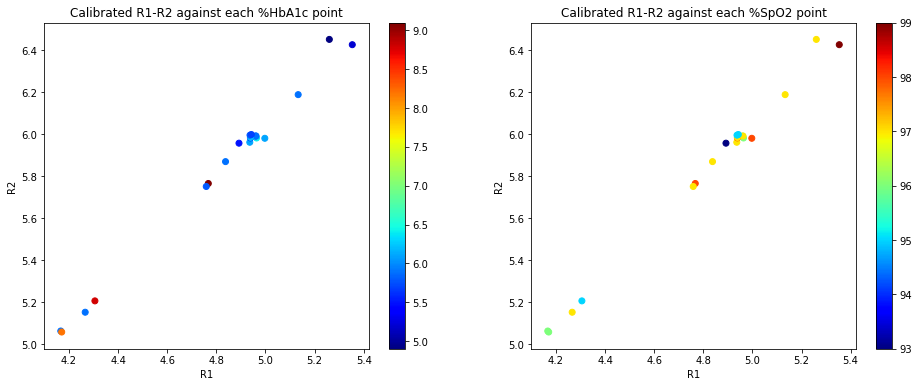


Figure 7: Calibrated R1-R2 for blood vessel model having calculated R1-R2 and BMI as input features.


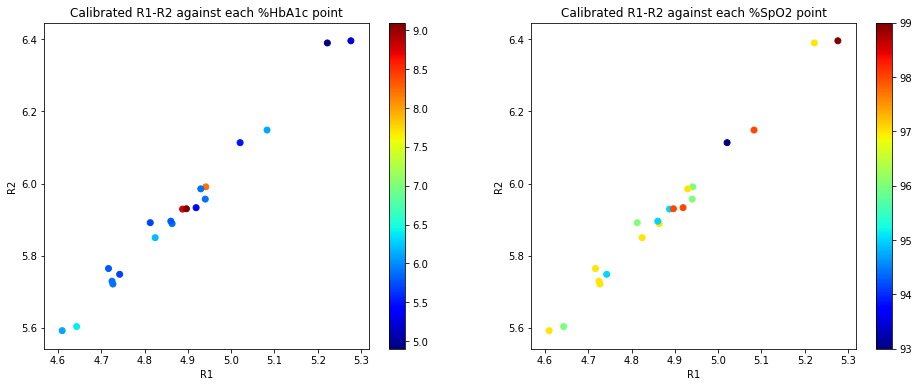


Figure 8: Calibrated R1-R2 for blood vessel model having calculated R1-R2 as input features only.

Comparing Figs. 5-8 with Fig. 1, we can see that the R1-R2 distribution of Fig. 1 is mostly identical with Fig. 5, and the R1-R2 points are gradually scattered as features decrease for the calibration model.

Similar analysis can also be performed on the whole finger model for different feature sets. The figures are provided in Fig. 9 to 12 below.


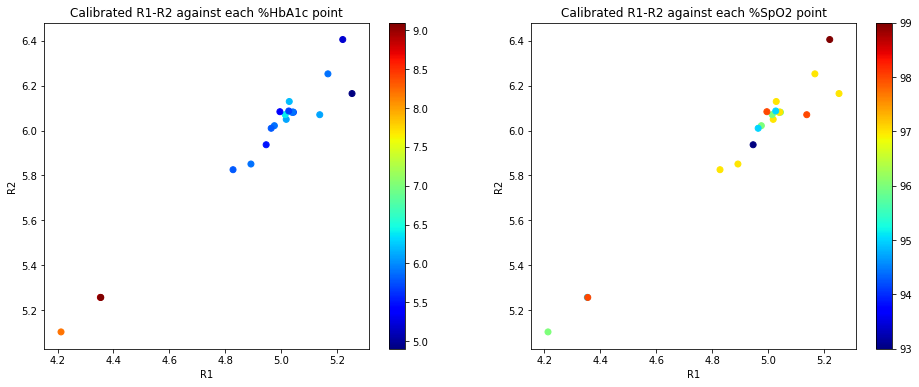


Figure 9: Calibrated R1-R2 for whole finger model having calculated R1-R2, finger width, and BMI as input features.


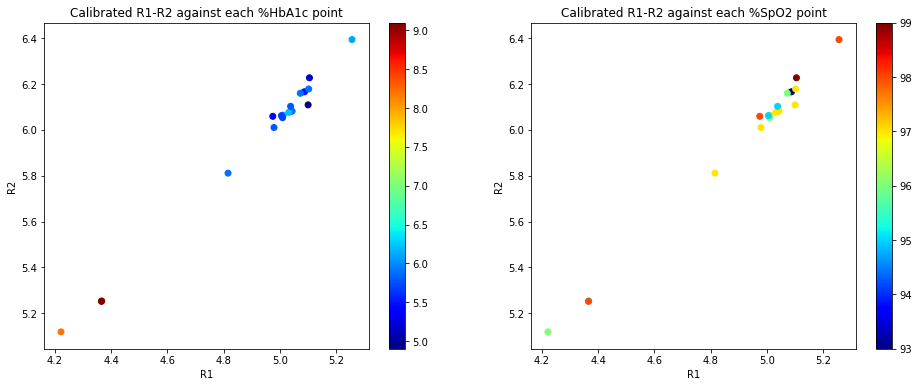


Figure 10: Calibrated R1-R2 for blood vessel model having calculated R1-R2 and finger width as input features.


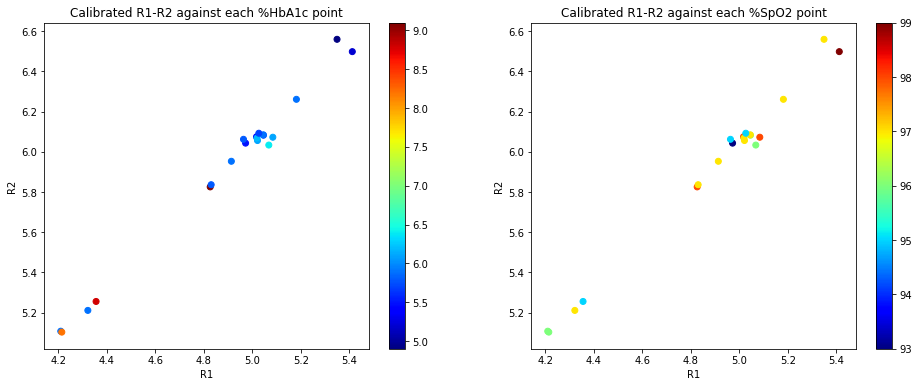


Figure 11: Calibrated R1-R2 for blood vessel model having calculated R1-R2 and BMI as input features.


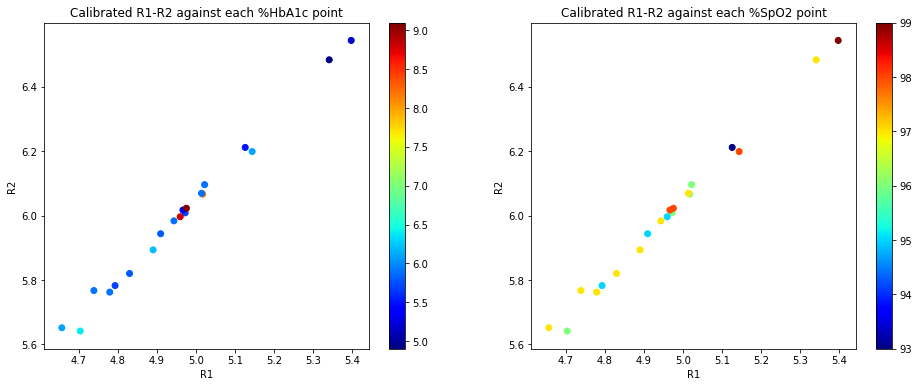


Figure 12: Calibrated R1-R2 for blood vessel model having calculated R1-R2 as input features only.

1. **Comparison of HbA1c results with Error Grid Analysis (EGA) and error metrics for different input features for ratio calibration models (Blood-vessel model):**

In this section, the two Pearson’s R (at two measurement points 1 and 2) results are shown in respect of HbA1c estimation metrics. The two measurement points are shown in Fig 13.


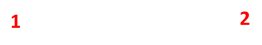

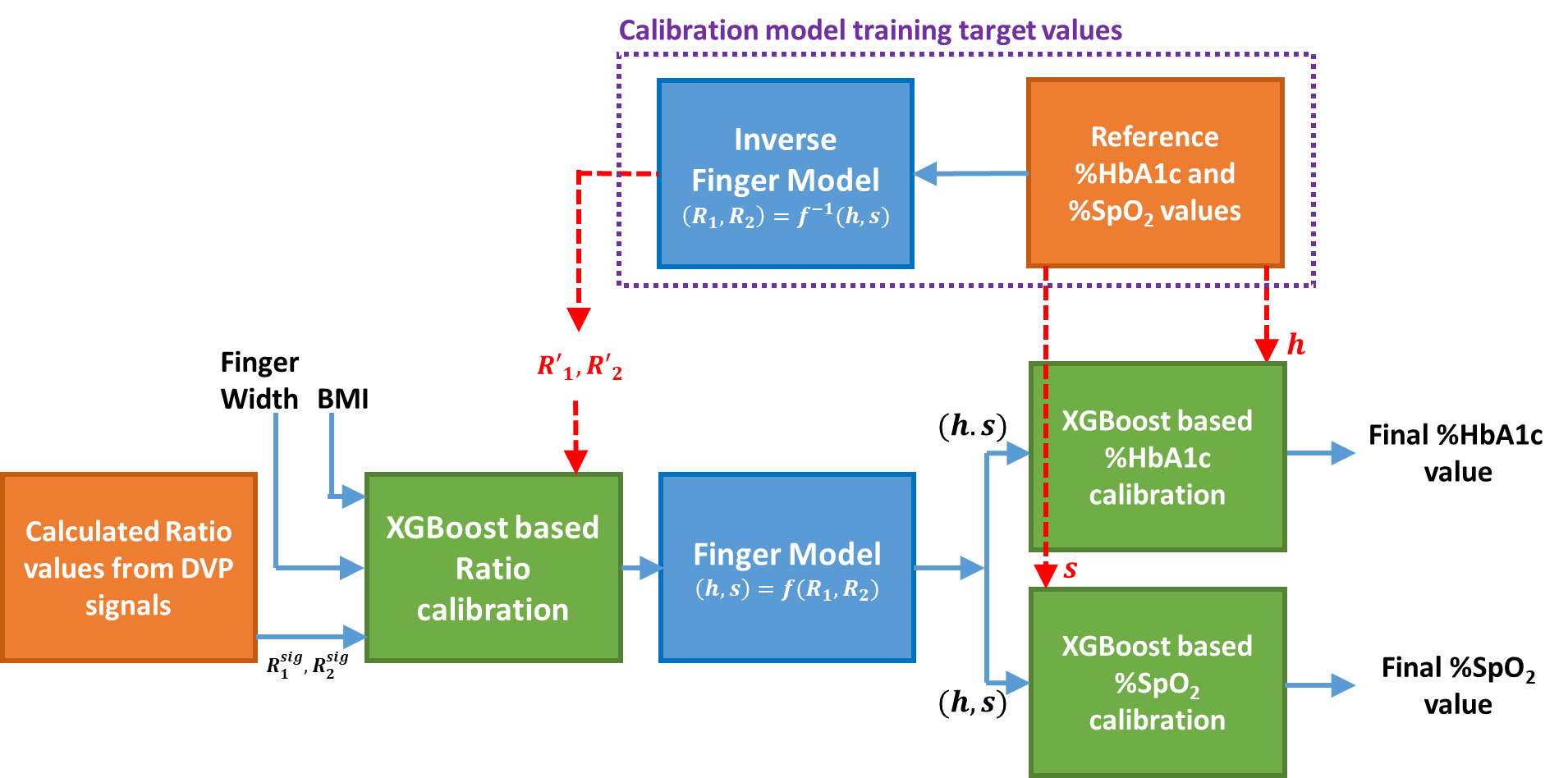


Figure 13: Calibration methodology. Points 1 and 2 are the two measurement points of estimated HbA1c values for quantitative analysis.

The calibration metrics of HbA1c at point 1 are calculated by estimating the HbA1c values from calibrated R1-R2 values. And the value calibration results are shown for HbA1c estimation at point 2 for the same calibrated R1-R2 values.

The EGA plots for these two different measurement points are depicted below for the blood vessel model (Fig. 14-17).

| 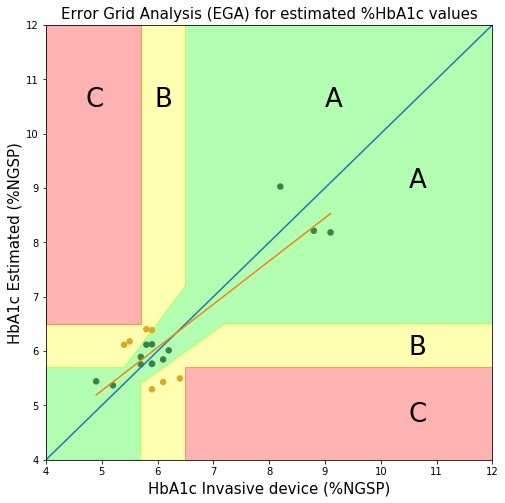 | 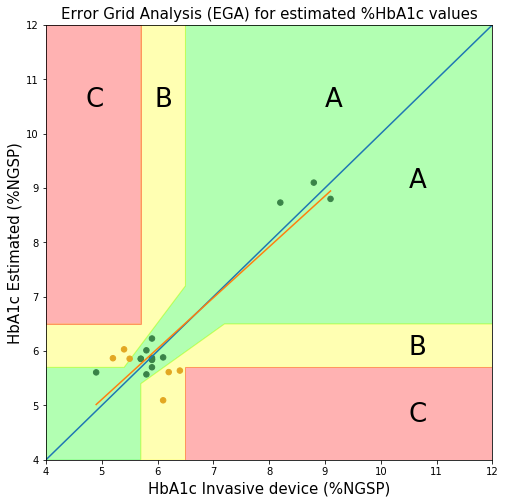 |
| --- | --- |
| (a) | (b) |

Figure 14: EGA plot for estimated HbA1c values (a) after ratio calibration (point 1) and (b) after %HbA1c value calibration (point 2). The inputs of ratio calibration model were R1-R2, finger width, and BMI.

| 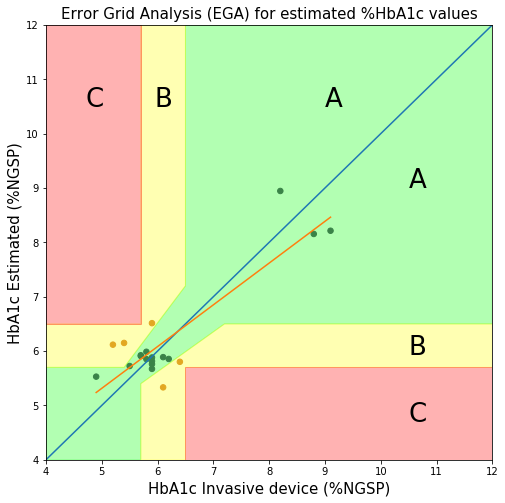 | 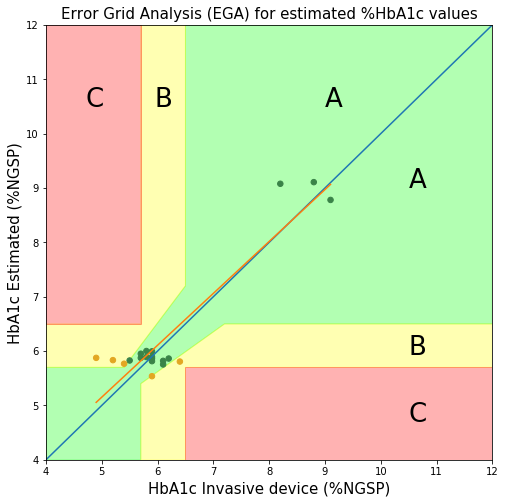 |
| --- | --- |
| (a) | (b) |

Figure 15: EGA plot for estimated HbA1c values (a) after ratio calibration (point 1) and (b) after %HbA1c value calibration (point 2). The inputs of ratio calibration model wereR1-R2 and finger width.

| 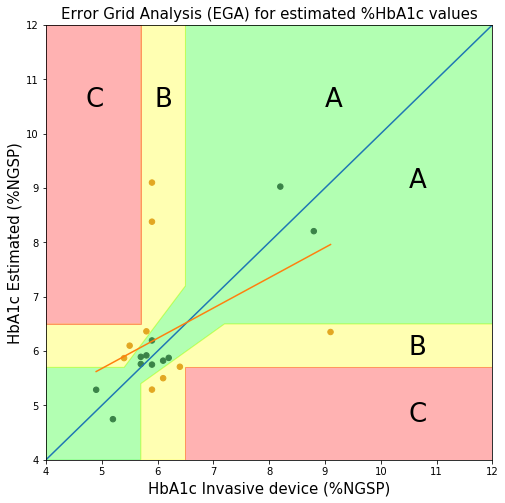 | 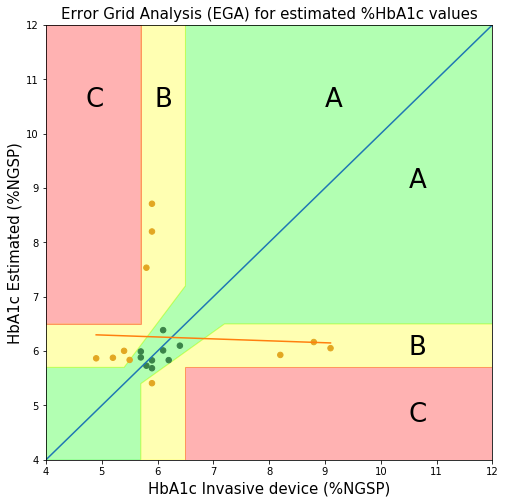 |
| --- | --- |
| (a) | (b) |

Figure 16: EGA plot for estimated HbA1c values (a) after ratio calibration (point 1) and (b) after %HbA1c value calibration (point 2). The inputs of ratio calibration model were R1-R2 and BMI.

| 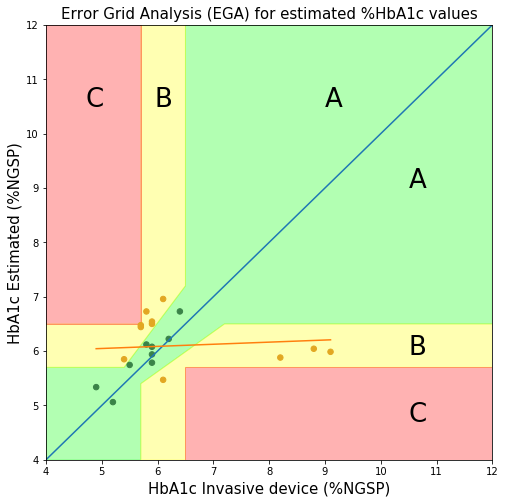 | 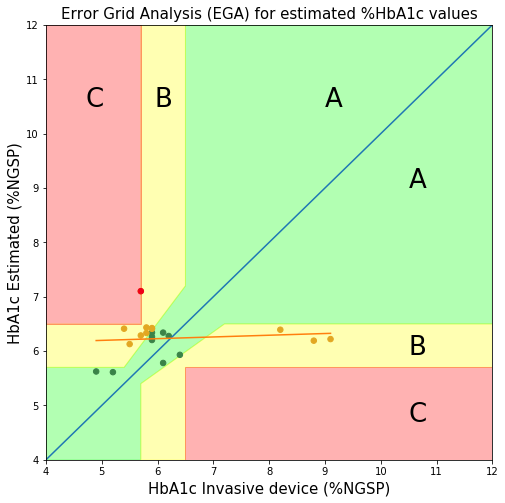 |
| --- | --- |
| (a) | (b) |

Figure 17: EGA plot for estimated HbA1c values (a) after ratio calibration (point 1) and (b) after %HbA1c value calibration (point 2). The inputs of ratio calibration model were only R1-R2.

Pearson’s R correlation metrics for these calibration setups are given below –

| **Measurement Point** | **R1-R2, FW, BMI** | **R1-R2, FW** | **R1-R2, BMI** | **R1-R2** |
| --- | --- | --- | --- | --- |
| **1** | 0.86 | 0.88 | 0.50 | 0.09 |
| **2** | 0.92 | 0.93 | -0.05 | 0.11 |

1. **Comparison of HbA1c results with Error Grid Analysis (EGA) and error metrics for different input features for ratio calibration models (Whole-finger model):**

Similar analysis can be done for the whole-finger model also. The comparison figures (Fig. 18 to 21) and evaluation metrics (Pearson’s R correlation coefficient) are given below.

| 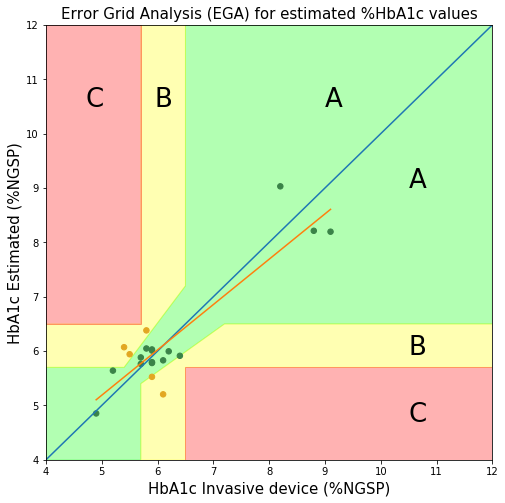 | 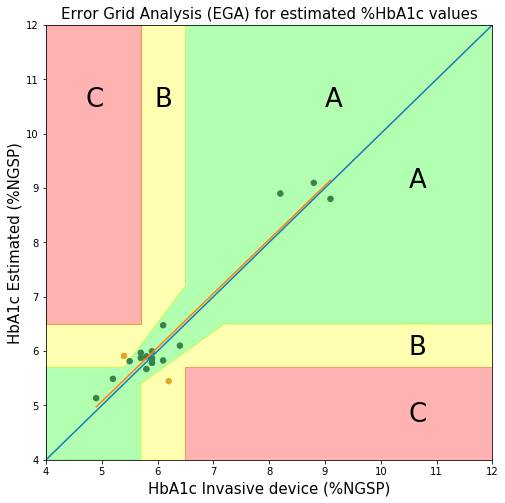 |
| --- | --- |
| (a) | (b) |

Figure 18: EGA plot for estimated HbA1c values (a) after ratio calibration (point 1) and (b) after %HbA1c value calibration (point 2). The inputs of ratio calibration model were R1-R2, finger width, and BMI.

| 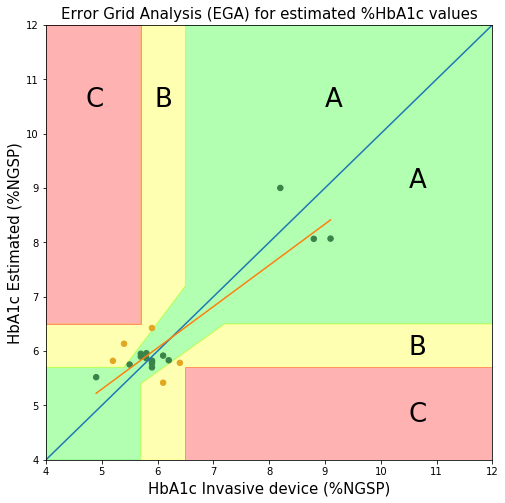 | 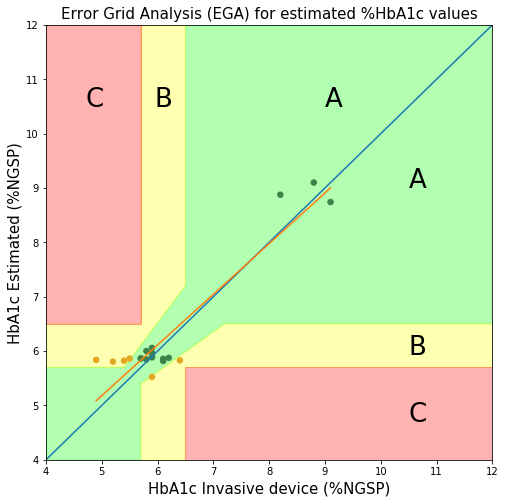 |
| --- | --- |
| (a) | (b) |

Figure 19: EGA plot for estimated HbA1c values (a) after ratio calibration (point 1) and (b) after %HbA1c value calibration (point 2). The inputs of ratio calibration model were R1-R2 and finger width.

| 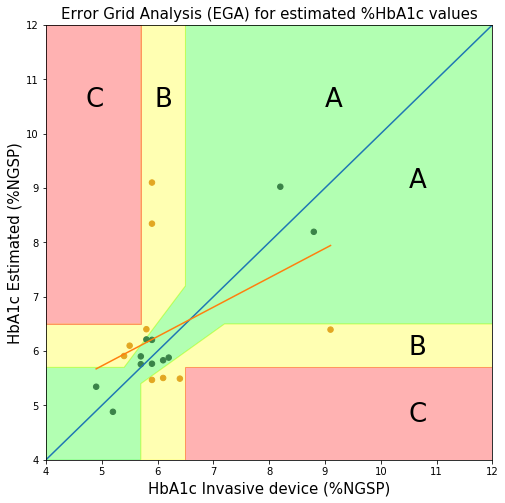 | 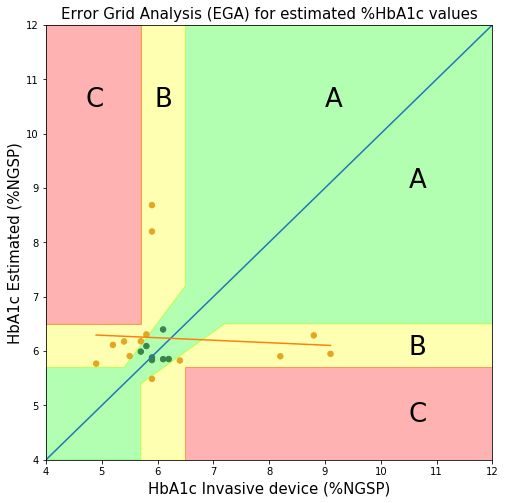 |
| --- | --- |
| (a) | (b) |

Figure 20: EGA plot for estimated HbA1c values (a) after ratio calibration (point 1) and (b) after %HbA1c value calibration (point 2). The inputs of ratio calibration model were R1-R2 and BMI.

| 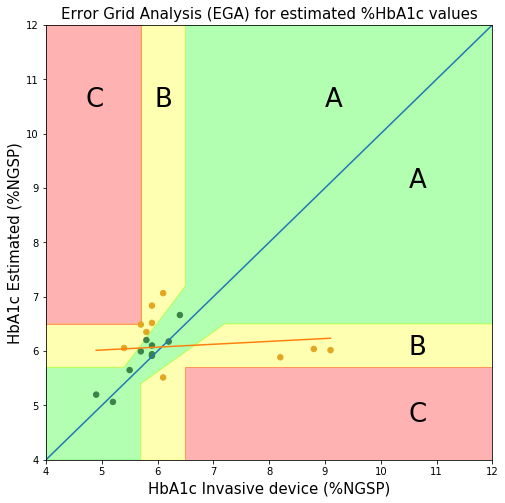 | 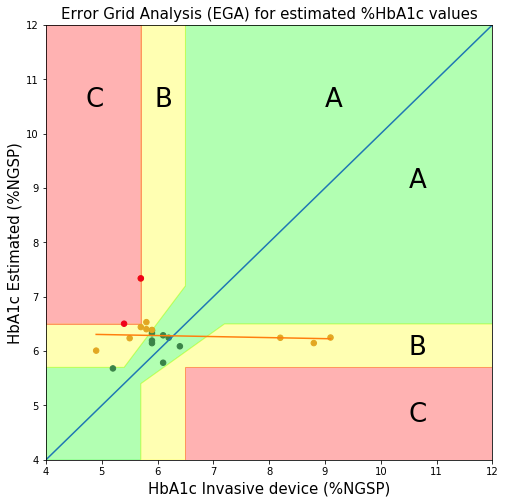 |
| --- | --- |
| (a) | (b) |

Figure 21: EGA plot for estimated HbA1c values (a) after ratio calibration (point 1) and (b) after %HbA1c value calibration (point 2). The inputs of ratio calibration model were only R1-R2.

Pearson’s R correlation metrics for these calibration setups are given below –

| **Measurement Point** | **R1-R2, FW, BMI** | **R1-R2, FW** | **R1-R2, BMI** | **R1-R2** |
| --- | --- | --- | --- | --- |
| **1** | 0.90 | 0.89 | 0.49 | 0.12 |
| **2** | 0.96 | 0.94 | -0.07 | -0.07 |

1. **Patient dataset Information:**

| **ID** | **Reference HbA1c**  **(%NGSP)** | **Reference SpO_2_**  **(%)** | **Age**  **(y)** | **Gender** | **Finger Width**  **(cm)** | **Height**  **(cm)** | **Weight**  **(kg)** | **BMI** |
| --- | --- | --- | --- | --- | --- | --- | --- | --- |
| 0 | 4.9 | 97 | 26 | F | 1.1 | 156 | 70 | 28.8 |
| 1 | 5.7 | 96 | 27 | M | 1.3 | 175 | 98 | 31.9 |
| 2 | 5.9 | 96 | 25 | M | 1.4 | 165 | 90 | 33.1 |
| 3 | 5.2 | 99 | 28 | F | 1.4 | 161 | 70 | 27.0 |
| 4 | 6.2 | 97 | 31 | M | 1.3 | 170 | 73 | 25.3 |
| 5 | 5.9 | 97 | 27 | M | 1.4 | 169 | 92 | 32.2 |
| 6 | 5.7 | 95 | 27 | M | 1.3 | 175 | 98 | 31.9 |
| 7 | 5.8 | 97 | 25 | M | 1.3 | 172 | 64 | 21.6 |
| 8 | 8.8 | 95 | 55 | M | 1.5 | 163 | 88 | 33.1 |
| 9 | 8.2 | 96 | 55 | M | 1.5 | 163 | 88 | 33.1 |
| 10 | 5.9 | 97 | 32 | M | 1.1 | 168 | 73 | 25.9 |
| 11 | 5.4 | 98 | 26 | F | 1.2 | 157 | 68 | 27.6 |
| 12 | 5.8 | 95 | 31 | M | 1.3 | 175 | 85 | 27.8 |
| 13 | 9.1 | 98 | 55 | M | 1.5 | 163 | 88 | 33.1 |
| 14 | 5.9 | 97 | 27 | M | 1.3 | 175 | 98 | 31.9 |
| 15 | 6.4 | 96 | 26 | F | 1.1 | 157 | 58 | 23.5 |
| 16 | 6.1 | 98 | 27 | M | 1.2 | 167 | 68 | 24.4 |
| 17 | 6.1 | 97 | 27 | M | 1.3 | 182 | 93 | 28.1 |
| 18 | 5.5 | 93 | 26 | F | 1.1 | 162 | 63 | 24.0 |
| 19 | 5.9 | 97 | 29 | M | 1.3 | 187 | 115 | 32.9 |

1. **Reference HbA1c device precision analysis:**

We conducted a precision analysis on the reference device with only four participants. For this test, three trials were performed with the reference invasive HbA1c device.

| **Reference HbA1c device precision test** | | | | | | |
| --- | --- | --- | --- | --- | --- | --- |
| **Patient ID** | **Trial 1** | **Trial 2** | **Trial 3** | **Mean** | **Standard Deviation** | **%CV** |
| 6 | 6.0 | **5.7** | 5.6 | 5.77 | 0.170 | 2.95 |
| 8 | **8.8** | 9.1 | 8.2 | 8.7 | 0.374 | 4.3 |
| 13 | **9.1** | 9.7 | 8.6 | 9.13 | 0.450 | 4.92 |
| T0 | 5.6 | **5.9** | 6.0 | 5.83 | 0.170 | 2.91 |

The HbA1c trial result closest to the mean of these trials was selected as each participant’s HbA1c value. The patient T0 participated only in the HbA1c device precision test and did not participate in this HbA1c and SpO_2_ estimation study.

Though this study comprises of very small test cohort and was not statistically significant, this was performed to account for the precision variability of invasive reference HbA1c devices over a specific range.
